# Evaluation of pneumococcal meningitis clusters in Burkina Faso and implications for potential reactive vaccination

**DOI:** 10.1016/j.vaccine.2020.06.002

**Published:** 2020-07-31

**Authors:** Heidi M. Soeters, Dinanibè Kambiré, Guetawendé Sawadogo, Rasmata Ouédraogo-Traoré, Brice Bicaba, Isaïe Medah, Lassana Sangaré, Abdoul-Salam Ouédraogo, Soumeya Ouangraoua, Issaka Yaméogo, Malika Congo-Ouédraogo, Absatou Ky Ba, Flavien Aké, Srinivasan Velusamy, Lesley McGee, Chris Van Beneden, Cynthia G. Whitney

**Affiliations:** a Centers for Disease Control and Prevention, Atlanta, GA, USA; b Centre Hospitalier Universitaire Pédiatrique Charles De Gaulle, Ouagadougou, Burkina Faso; c Davycas International, Ouagadougou, Burkina Faso; d Ministère de la Santé, Ouagadougou, Burkina Faso; e Centre Hospitalier Universitaire-Yalgado Ouédraogo, Ouagadougou, Burkina Faso; f Centre Hospitalier Universitaire Sourou Sanou, Bobo-Dioulasso, Burkina Faso; g Centre Muraz, Bobo-Dioulasso, Burkina Faso; h Laboratoire National de Santé Publique, Ouagadougou, Burkina Faso

**Keywords:** Burkina Faso, Pneumococcal meningitis, Pneumococcal conjugate vaccine, Outbreaks, Surveillance

## Abstract

**Background:**

To better understand how to prevent and respond to pneumococcal meningitis outbreaks in the meningitis belt, we retrospectively examined Burkina Faso’s case-based meningitis surveillance data for pneumococcal meningitis clusters and assessed potential usefulness of response strategies.

**Methods:**

Demographic and clinical information, and cerebrospinal fluid laboratory results for meningitis cases were collected through nationwide surveillance. Pneumococcal cases were confirmed by culture, polymerase chain reaction (PCR), or latex agglutination; strains were serotyped using PCR. We reviewed data from 2011 to 2017 to identify and describe clusters of ≥ 5 confirmed pneumococcal meningitis cases per week in a single district. We assessed whether identified clusters met the 2016 WHO provisional pneumococcal meningitis outbreak definition: a district with a weekly incidence of >5 suspected meningitis cases/100,000 persons, >60% of confirmed meningitis cases caused by *Streptococcus pneumoniae*, and >10 confirmed pneumococcal meningitis cases.

**Results:**

Twenty pneumococcal meningitis clusters were identified, with a maximum weekly incidence of 7 cases and a maximum duration of 4 weeks. Most identified clusters (15/20; 75%) occurred before nationwide introduction of 13-valent pneumococcal conjugate vaccine (PCV13) in October 2013. Most cases were due to serotype 1 (74%), 10% were due to PCV13 serotypes besides serotype 1, and 8 clusters had >1 serotype*.* While 6 identified clusters had a weekly incidence of >5 suspected cases/100,000 and all 20 clusters had >60% of confirmed meningitis cases due to *S. pneumoniae*, no cluster had >10 confirmed pneumococcal meningitis cases in a single week.

**Conclusions:**

Following PCV13 introduction, pneumococcal meningitis clusters were rarely detected, and none met the WHO provisional pneumococcal outbreak definition. Due to the limited cluster size and duration, there were no clear instances where reactive vaccination could have been useful. More data are needed to inform potential response strategies.

## Highlights

• From 2011 to 2017, Burkina Faso had 20 pneumococcal meningitis clusters of ≥ 5 cases per district/week.
• Clusters had a maximum weekly incidence of 7 cases and a maximum duration of 4 weeks.
• Most clusters occurred prior to 13-valent pneumococcal conjugate vaccine introduction.
• Clusters were caused by a mixture of serotypes, with serotype 1 being most common.
• Due to the limited cluster size and duration, there were no clear indications for reactive vaccination.

## 1 Introduction

*Streptococcus pneumoniae* is a leading infectious cause of global morbidity and mortality [1] and a primary etiology of bacterial meningitis, along with *Neisseria meningitidis* and *Haemophilus influenzae* [2]. In 2017, an estimated 445,000 cases of pneumococcal meningitis with 42,000 deaths occurred globally [3]. In the meningitis belt of sub-Saharan Africa, pneumococcal meningitis has a distinct seasonality similar to that of meningococcal meningitis, high case fatality ratio (CFR), and predominance of serotype 1 disease in persons aged >5 years [4], [5], [6], [7], [8], [9].

Burkina Faso is a West African country that is located entirely within the meningitis belt and experiences hyper-endemic rates of meningitis [10]. After the successful introductions of the *H. influenzae* serotype b (Hib) vaccine in 2006 [11] and the meningococcal serogroup A conjugate vaccine (MACV, MenAfriVac®) in 2010 [12], [13], *S. pneumoniae* became the predominant cause of the remaining bacterial meningitis burden in Burkina Faso. The government of Burkina Faso introduced 13-valent pneumococcal conjugate vaccine (PCV13) into the routine childhood immunization program in October 2013 using a schedule with 3 primary doses given at 8, 12, and 16 weeks of age without a booster or catch-up campaign. The WHO-UNICEF estimate of vaccination coverage with 3 doses of PCV13 in Burkina Faso was 91% for 2014–2017 [14]. Burkina Faso is one of the few African countries to both successfully implement nationwide case-based meningitis surveillance and to also routinely serotype all pneumococcal meningitis specimens, in an effort to evaluate PCV13 impact [9], [15], [16], [17], [18].

Before PCV13 introduction in Burkina Faso, the highest pneumococcal meningitis incidence and mortality occurred among children aged < 1 year, and 71% of cases were due to PCV13 serotypes [18]. In the first 4 years after nationwide PCV13 introduction, meningitis due to PCV13-serotypes declined substantially (62%), both among vaccinated age groups (children aged < 1 year: 77% reduction) and among older age groups potentially benefitting from herd protection (persons aged ≥ 15 years: 64% reduction) [15], [16]. However, the decline in incidence was larger for PCV13 serotypes excluding serotype 1 (79% reduction) than for serotype 1 (52% reduction). These data suggested impact of PCV13, but that efforts to improve control of serotype 1, such as switching from a 3 + 0 schedule to a 2 + 1 schedule, may improve overall control of pneumococcal meningitis in this setting [15], [19].

Numerous countries in sub-Saharan Africa, including neighboring Ghana, struggle to control pneumococcal meningitis burden and outbreaks, even in the context of high coverage with pneumococcal conjugate vaccine (PCV) [8], [20], [21], [22], [23], [24], [25]. Unlike for meningococcal meningitis, there is no formal epidemic definition or outbreak response guidelines for pneumococcal meningitis [26]. These experiences prompted discussions about potential methods to prevent and respond to pneumococcal meningitis outbreaks in the meningitis belt [27]. To help inform these discussions, we retrospectively examined Burkina Faso’s case-based meningitis surveillance data to identify and describe pneumococcal meningitis clusters. These data help us understand pneumococcal meningitis epidemiologic dynamics that may be hidden within the predominant context of meningococcal meningitis in this region. We also examined the trajectories of identified clusters to assess whether vaccination or other response strategies could have potentially been used to decrease the spread or duration of the identified clusters.

## 2 Materials and methods

### 2.1 National surveillance system

Burkina Faso has collected high-quality case-based meningitis surveillance data nationwide since 2010 [17], [18]. Case-level demographic and clinical information, as well as cerebrospinal fluid (CSF) specimens, were collected from all suspected meningitis cases in all districts using WHO and MenAfriNet instruments [28], [29]; specimens were tested at 5 national reference laboratories. From 2011 to 2015, Burkina Faso had 63 districts total; following re-districting, this number changed to 70 districts in 2016 and 2017.

According to WHO case definitions [30], a suspected meningitis case is sudden onset of fever ≥ 38.5 °C with neck stiffness, altered consciousness, or other meningeal signs (including flaccid neck, bulging fontanel, or convulsions in young children). A laboratory-confirmed pneumococcal meningitis case is a suspected case with *S. pneumoniae* isolated from CSF by culture or detected in CSF by real-time polymerase chain reaction (PCR) targeting the *lytA* gene or latex agglutination, using laboratory methods previously described [18], [31]. In the case of discrepant results for case confirmation, methods were considered confirmatory in the following order: PCR, culture, and latex agglutination. Serotyping of confirmed pneumococcal cases was performed using a multiplex real-time PCR approach targeting 37 serotypes; this method detects all 13 vaccine serotypes but is unable to differentiate some genetically similar serotypes (e.g., 6A/6B, 12F/12A/12B/44/46) [32]. Not all pneumococci were serotyped, as some cases were only confirmed via latex agglutination, and some did not have a specimen or isolate available for serotyping or had an insufficient quantity available for serotyping.

### 2.2 Statistical methods

We retrospectively analyzed pneumococcal meningitis cases diagnosed from January 1, 2011 to December 31, 2017. Cases in non-residents of Burkina Faso (n = 56) were excluded from the analyses. Pneumococcal meningitis cases were categorized as due to PCV13 serotypes (1, 3, 4, 5, 6A/6B, 7F/7A, 9 V/9A, 14, 18C/18F/18B/18A, 19A, 19F, or 23F), non-PCV13 serotypes, or non-typeable specimens [18]. The numbers of suspected, pneumococcal, meningococcal, and *H. influenzae* meningitis cases reported per district per WHO epidemiologic week (i.e., a district-week) were calculated. Crude annual incidences (cases per 100,000 persons) were calculated using district-level population census estimates projected from the 2006 census. In 2017, Burkina Faso had a total population of 19,632,147 and an estimated birth cohort of 762,074 [33]. The CFRs were calculated by dividing the number of reported deaths by the total number of cases.

For the purposes of this analysis, a cluster was defined as ≥ 5 confirmed pneumococcal meningitis cases reported in a district in one WHO epidemiologic week. For each identified cluster, we reviewed the case counts, incidence, serotypes, age distribution, and other detected pathogens for all suspected meningitis cases reported in that district during the year of the cluster. We described some key illustrative clusters. We also compared the weekly incidence of suspected cases to thresholds of ≥ 10, ≥ 5, or ≥ 3 cases per 100,000 population, to see whether any of these potential alert thresholds could have detected these pneumococcal meningitis clusters in “real time”. We also compared the clusters to the 2016 WHO provisional pneumococcal meningitis outbreak definition, which includes 3 required elements: 1) a district or subdistrict with a weekly incidence of >5 suspected cases per 100,000 population; 2) >60% of confirmed meningitis cases caused by *S. pneumoniae*; and 3) >10 confirmed cases of pneumococcal meningitis [26].

### 2.3 Study approval

This analysis was approved by Burkina Faso Ministry of Health ethical committee and was determined by the Centers for Disease Control and Prevention’s (CDC) Human Research Protection Office to be public health non-research. As this analysis involved routinely-collected surveillance data, informed consent was not required. Surveillance data were anonymized to maintain patient privacy and confidentiality.

## 3 Results

### 3.1 Pneumococcal meningitis surveillance data

From 2011 to 2017, 23,705 suspected meningitis cases were reported in Burkina Faso (Table 1). CSF was collected in almost all cases (98%), and 15,193 (64%) of CSF specimens were tested via latex, culture, or PCR. In total, 3498 (15%) suspected meningitis cases were laboratory-confirmed as *S. pneumoniae*. The majority (87%) of pneumococcal meningitis cases were confirmed using PCR, with or without another positive test (see Supplemental Table). In total, 2652 (76%) pneumococcal specimens were serotyped. Among these, 2156 (81%) had a serotype and 496 (19%) were non-typeable. The proportion of pneumococcal meningitis cases that were serotyped increased from 60% in 2011 to 86% in 2017. The CFR among all pneumococcal meningitis cases was 20%.

**Table 1:** Meningitis cases, Burkina Faso, 2011–2017.

	**Pre-PCV13 introduction**						**Post-PCV13 introduction**									
	**2011**		**2012**		**2013**		**2014**		**2015**		**2016**		**2017**		**Total**	
	**N (%)**		**N (%)**		**N (%)**		**N (%)**		**N (%)**		**N (%)**		**N (%)**		**N (%)**	
Suspected meningitis cases	2841		6499		2829		3399		2970		2659		2508		23,705	
CSF collected	2766	(97)	6302	(97)	758	(97)	3331	(98)	2912	(98)	2603	(98)	2474	(99)	23,146	(98)
CSF tested^a^	1718	(60)	2894	(45)	2011	(71)	2312	(68)	2229	(75)	2140	(80)	1889	(75)	15,193	(64)
Pneumococcal meningitis cases^b^	642		462		424		502		551		520		397		3498	
Confirmed using PCR	474	(74)	353	(76)	383	(90)	447	(89)	520	(94)	487	(94)	370	(93)	3034	(87)
Serotyped	384	(60)	326	(71)	326	(77)	390	(78)	487	(88)	397	(76)	342	(86)	2652	(76)
PCV13 serotypes	291	(76)	234	(72)	212	(65)	298	(76)	340	(70)	244	(62)	194	(57)	1813	(68)
Non-PCV13 serotypes	30	(8)	49	(15)	62	(19)	53	(14)	47	(10)	59	(15)	43	(13)	343	(13)
Nontypeable specimens	63	(16)	43	(13)	52	(16)	39	(10)	100	(21)	94	(24)	105	(31)	496	(19)
Reported deaths	179	(28)	94	(20)	84	(20)	99	(20)	101	(18)	90	(17)	49	(12)	696	(20)

Note: Some of the data presented in this table have been previously published [15].

Abbreviations: CSF, cerebrospinal fluid; PCR, polymerase chain reaction; PCV13, 13-valent pneumococcal conjugate vaccine.

a Tested by culture, PCR, or latex agglutination.

b *S. pneumoniae* isolated from CSF by culture or detected in CSF by PCR or latex agglutination.

### 3.2 Pneumococcal meningitis cluster identification

**Fig. 1:**
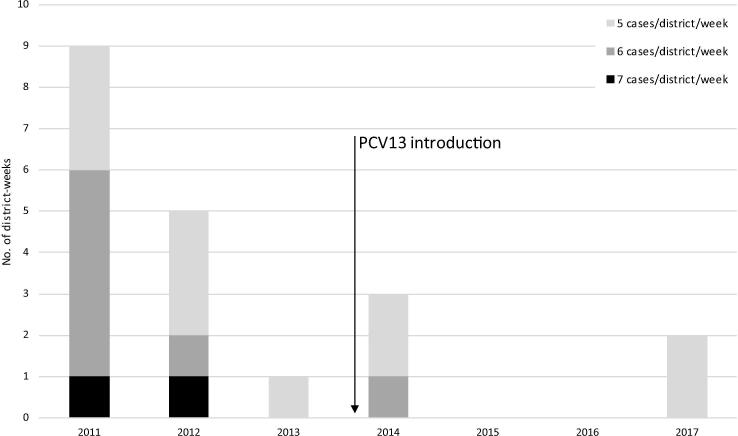
District-level epidemiologic weeks (i.e. district-weeks) with ≥ 5 pneumococcal meningitis cases reported in a district in one week, Burkina Faso, 2011–2017. Note: In 2012, epidemiologic weeks 14, 15, and 16 in the district of Koudougou made up 3/5 of the district-weeks with ≥ 5 pneumococcal meningitis cases. Similarly, in 2014, epidemiologic weeks 2, 3, and 4 in the district of Houndé made up all 3 of the district-weeks with ≥ 5 pneumococcal meningitis cases.

Burkina Faso was comprised of 63 districts from 2011 to 2015 and 70 districts in 2016–2017, resulting in a total of 23,723 district-level epidemiologic weeks (i.e., district-weeks from 2011 to 2017). In 20 (0.08%) of these district-weeks, ≥5 confirmed pneumococcal meningitis cases were reported in a district in a single week, indicating a pneumococcal meningitis cluster. In 11 (55%) of these clusters, 5 pneumococcal meningitis cases were reported in that district-week; 7 (35%) of the clusters consisted of 6 pneumococcal meningitis cases; and 2 (10%) of the clusters had 7 pneumococcal meningitis cases (Fig. 1). No districts in Burkina Faso reported >7 pneumococcal meningitis cases in a single week during 2011–2017.

### 3.3 Pneumococcal meningitis cluster description

Most of the identified clusters (15/20; 75%) occurred prior to PCV13 introduction in October 2013 (Fig. 1). Nearly half of clusters (9/20; 45%) were reported in 2011; these clusters varied geographically and temporally, occurring in 8 different districts and in epidemiologic weeks 3 to 11 (Table 2). In 2011, only 1 district (Kaya) reported ≥ 5 pneumococcal meningitis cases in 2 different though consecutive weeks (weeks 10 and 11). In 2012, three of the districts-weeks with ≥ 5 reported pneumococcal meningitis cases were in fact part of a larger cluster that occurred in the same district (Koudougou) in consecutive weeks (weeks 14, 15, and 16). Similarly, in 2014, all three of the district-weeks with ≥ 5 cases occurred in Houndé in weeks 2, 3, and 5 (also, 3 cases occurred in week 4). No clusters were detected in 2015 or 2016. In 2017, 2 districts (Kaya and Titao) had clusters of 5 cases each.

**Table 2:** Retrospectively identified pneumococcal meningitis clusters defined as ≥ 5 confirmed pneumococcal meningitis cases reported in a district in one week, Burkina Faso, 2011–2017.

**Year**	**District**	**Epidemiologic Week**	**No. suspected cases**	**Suspected case incidence (per 100,000)**	**No. pneumococcal meningitis cases**^a^	**No. lab-confirmed cases**^b^	**Proportion of all lab-confirmed cases**^b^**that week due to *S. pneumoniae***
2011	Dafra	6	12	4.4	6	6	100%
2011	Houndé	8	9	3.4	6	7	86%
2011	Karangasso Vigué	8	10	11.0	6	6	100%
2011	Kaya	10	9	1.7	6	7	86%
2011	Kaya	11	7	1.4	5	5	100%
2011	Ouahigouya	9	10	2.3	6	6	100%
2011	Pama	8	10	10.6	5	7	71%
2011	Saponé	3	6	6.4	5	5	100%
2011	Tenkodogo	7	8	4.0	7	8	88%
2012	Dori	10	7	2.3	5	6	83%
2012	Koudougou	14	12	2.7	6	7	86%
2012	Koudougou	15	9	2.1	7	7	100%
2012	Koudougou	16	12	2.8	5	5	100%
2012	Ouahigouya	15	42	9.2	5	5	100%
2013	Boromo	10	6	2.3	5	5	100%
2014	Houndé	2	29	9.8	5	5	100%
2014	Houndé	3	19	6.4	5	6	83%
2014	Houndé	5	11	3.7	6	6	100%
2017	Kaya	7	11	2.8	5	7	71%
2017	Titao	4	5	2.6	5	5	100%

a *S. pneumoniae* isolated from CSF by culture or detected in cerebrospinal fluid (CSF) by polymerase chain reaction (PCR) or latex agglutination.

b *S. pneumoniae*, *N. meningitidis*, or *H. influenzae* isolated from CSF by culture or detected in CSF by PCR or latex agglutination.

**Fig. 2:**
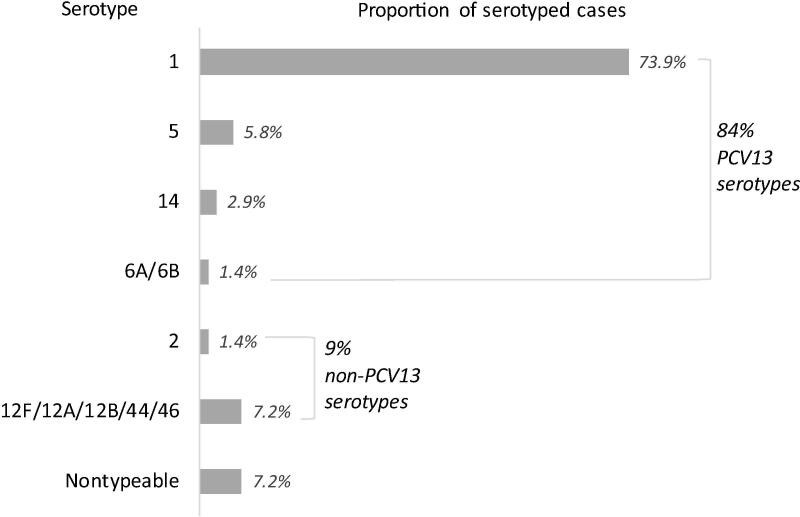
Serotype distribution of pneumococcal meningitis cases occurring in districts with ≥ 5 pneumococcal meningitis cases reported in one week, Burkina Faso, 2011–2017.

Of the 111 pneumococcal meningitis cases comprising the 20 clusters, 69 (62%) were serotyped. Of those, 51 (74%) were serotype 1, 10% were PCV13 serotypes besides serotype 1, 9% were non-PCV13 serotypes, and 7% were nontypeable (Fig. 2). Serotype 1 was over-represented among cluster-associated cases, as 49% of all serotyped pneumococcal meningitis cases occurring in Burkina Faso in 2011–2017 were serotype 1 (*P* < 0.01). However, the serotype 1 cases often occurred among a background of meningitis cases due to other pneumococcal serotypes, meningococcal serogroups, and *H. influenzae* (see Supplemental Material for detailed descriptions of each cluster). During the weeks when a district reported ≥ 5 pneumococcal meningitis cases, the proportion of laboratory-confirmed meningitis cases in that district that were due to pneumococcus ranged from 71% to 100% (median: 100%, Table 1).

The age distribution of cluster-associated cases (median: 9 years, interquartile range [IQR]: 4–13 years) did not significantly differ from non-cluster-associated pneumococcal meningitis cases (median: 10 years, IQR: 4–17 years; Wilcoxon *P* = 0.16).

### 3.4 Example clusters

#### 3.4.1 Koudougou 2012

In 2012, the district of Koudougou (population 433,545) reported ≥ 5 pneumococcal meningitis cases in each of 3 consecutive weeks (weeks 14, 15, and 16; see Supplemental Material). During these 3 weeks, 33 suspected meningitis cases were reported; 19 were laboratory-confirmed (18 *S. pneumoniae*, 1 *H. influenzae*). The pneumococcal cases were reported from 14 different villages; 6/18 cases were serotyped, and all were serotype 1. Following this 3-week-long cluster, 1 pneumococcal meningitis case was reported in week 17 and none were reported during the next 5 weeks. The highest suspected case incidence occurred during weeks 14 and 16 (2.8 per 100,000).

#### 3.4.2 Houndé 2014

In 2014, the district of Houndé (population 294,865) reported ≥ 5 pneumococcal meningitis cases in 3 nearly consecutive weeks (weeks 2, 3, and 5; 3 cases were reported in week 4; see Supplemental Material for more detail). During weeks 2–5, 72 suspected meningitis cases were reported; 21 were laboratory-confirmed (19 *S. pneumoniae*, 2 *N. meningitidis*). Most pneumococcal cases were reported from 4 villages. In total, 14/19 cases were serotyped: 11 (79%) were serotype 1, 2 were serotype 5, and 1 was serotype 12F/12A/12B/44/46. Following this 4-week-long cluster, 11 pneumococcal meningitis cases due to 6 different serotypes were reported during the next 8 weeks. The highest suspected case incidence occurred during week 2 (9.8 per 100,000).

#### 3.4.3 Comparison against potential alert thresholds and 2016 WHO provisional pneumococcal meningitis outbreak definition

For each of the 20 clusters, we compared the incidence of suspected cases to thresholds of ≥ 10, ≥ 5, or ≥ 3 cases per 100,000, to see whether any of these potential alert thresholds could have detected these pneumococcal meningitis clusters in “real time”. Two of the 20 clusters had a suspected case incidence of ≥ 10 cases per 100,000: Karangasso Vigué and Pama, both in 2011 (Table 2). Six of the clusters had a suspected case incidence of ≥ 5 cases per 100,000, and 10 had an incidence of ≥ 3 cases per 100,000.

None of the 20 clusters met the 2016 WHO provisional pneumococcal meningitis outbreak definition. While 6 clusters had a weekly incidence of >5 suspected cases per 100,000 and all 20 clusters had >60% of confirmed meningitis cases due to *S. pneumoniae*, none of the clusters had >10 confirmed cases of pneumococcal meningitis in a single week.

## 4 Discussion

This retrospective analysis of meningitis surveillance data from Burkina Faso identified 20 pneumococcal meningitis clusters of ≥ 5 cases reported in a district in a single week. However, none of these clusters met the 2016 WHO provisional pneumococcal meningitis outbreak definition. Though the identified clusters often included a mixture of pneumococcal serotypes, serotype 1 caused the majority (74%) of cases. Additionally, as most clusters were of short duration, there were no clear cases where reactive vaccination with PCVs might have been useful.

Though currently in sub-Saharan Africa, PCVs are currently only used in the context of routine childhood immunization via the Expanded Programme on Immunization, the WHO has suggested that pneumococcal vaccines could be used in mass vaccination for children and adults in outbreaks of pneumococcal meningitis, in a similar way to that used in outbreaks of meningococcal meningitis [26]. However, there are a number of challenges when considering the potential role of PCVs for reactive vaccination. First, the effectiveness of reactive vaccination in reducing further cases of pneumococcal meningitis is not known [26]. Reactive vaccination of districts or sub-districts requires an immense mobilization effort, so a clear understanding of the benefits is a prerequisite. Second, unlike the vaccines stockpiled for meningococcal meningitis outbreak response, the WHO’s International Coordinating Group (ICG) on Vaccine Provision [34], [35] does not have a stockpile of PCVs. Third, laboratory confirmation of *S. pneumoniae* and the corresponding serotypes is not done in real-time in Burkina Faso. Unlike for meningococcal meningitis, where initial latex agglutination testing can give public health authorities an idea of serogroup and where serogroup confirmation can sometimes be performed by regional laboratories, pneumococcal serotyping is often performed in batches at the national level and results are only available and linked to epidemiologic data after significant delay. Fourth, when reactive vaccination was used in response to recent meningococcal meningitis outbreaks in the meningitis belt, the median response time was over 5 weeks (median: 36 days; range: 10–64 days) [36] and an additional 2 weeks are assumed to be needed for development of an immune response, for a total of approximately 7 weeks before a vaccination response may begin to affect the trajectory of an outbreak. Therefore, by the time the necessary epidemiologic and laboratory information is pieced together and reactive PCV vaccination could be mobilized, it would likely be too late to produce any public health impact.

The two largest identified clusters occurred in the districts of Koudougou in 2012 and Houndé in 2014. In Koudougou, 18 confirmed pneumococcal meningitis cases occurred over 3 weeks; all serotyped cases were serotype 1. However, the cases occurred in many different villages, the suspected case incidence never crossed any potential alert thresholds, and there was likely no time for reactive vaccination to potentially play a role in public health response, as the cluster ended after 3 weeks. In Houndé, 19 confirmed pneumococcal meningitis cases occurred over 4 weeks. The cases were mainly concentrated in 4 villages, and the district reached a suspected case incidence of 9.8 cases per 100,000 in the first week of the cluster. Though this incidence crossed the 2016 WHO provisional outbreak definition of >5 suspected cases per 100,000, it did not cross the currently-used alert threshold of 10 suspected cases per 100,000. Additionally, the cases were due to a variety of serotypes, though most were covered by PCV13. In this instance, there also likely would not have been time to implement a reactive vaccination campaign, and it may not have been pursued anyway due to the variety of serotypes involved.

In the absence of reactive vaccination, few effective public health prevention or response measures are available to control pneumococcal meningitis outbreaks once they start. Antibiotic chemoprophylaxis for household contacts is not recommended during pneumococcal meningitis outbreaks, as household members are not considered at increased risk and antibiotic prophylaxis is not known to be effective [26]. Fortunately, we found fewer pneumococcal meningitis clusters in Burkina Faso after the nationwide PCV13 program was implemented, suggesting that the current infant vaccination program is helpful for preventing clusters.

One potential prevention tool that has been discussed to further reduce pneumococcal meningitis is to strive for better control of all invasive pneumococcal infections by adding a booster dose to the infant vaccination series [15], [37]. The WHO currently recommends both PCV booster-containing schedules (2 primary doses and 1 booster dose at 9–12 months of age: 2 + 1) and non-booster-containing schedules (3 primary doses: 3 + 0) [38]. Most Gavi-eligible countries and all African countries except South Africa and Mozambique use a 3 + 0 schedule, as WHO initially recommended the 3 + 0 schedule, with the 2 + 1 schedule being an acceptable alternative [39]. Many countries also implemented the 3 + 0 schedule for logistical harmonization with other infant immunizations and to maximize vaccination coverage. However, more recent data indicate that the 2 + 1 PCV schedule induces higher antibody levels in the second year of life [19], [38]. A booster dose could also lead to a longer duration of protection and improved effectiveness against serotype 1 disease [39], and may help prevent PCV13-serotype pneumococcal carriage among toddlers, who are likely the primary source of pneumococcal transmission in the community [19], [40], [41], [42]. Based on these data, Burkina Faso is considering changing from a 3 + 0 to a 2 + 1 schedule, which would involve removing one of the 3 primary doses (the middle dose, given at age 12 weeks) and adding a booster dose at the 9–12 month visit along with the first dose of measles-containing vaccine. An additional prevention strategy that could be considered is a catch-up campaign for older children, given that the median age of cases remained 8–11 years both pre- and post-PCV13 introduction.

This analysis was an exploratory review of retrospective data, to see if any lessons could be gleaned to guide conversations around prevention and response of pneumococcal meningitis outbreaks in the meningitis belt. Though it adds to the relatively limited literature base on this topic, there are limitations to this analysis. First, as with any surveillance system, there is a possibility that not all pneumococcal meningitis cases were detected, diagnosed, and reported. Additionally, though much effort has gone into the serotyping of pneumococcal meningitis in Burkina Faso and the proportion of specimens serotyped increased from 60% in 2011 to 86% in 2017, not all cases were serotyped. Furthermore, this analysis relied on pneumococcal meningitis surveillance data; a greater understanding of pneumococcal transmission dynamics would require data on other clinical presentations of invasive pneumococcal disease, non-invasive presentations, and nasopharyngeal carriage. While these elements have been a focus of recent work in Burkina Faso, they are not factored into this retrospective analysis. Another limitation is one previously mentioned by Coldiron et al [43] regarding the 2016 WHO provisional pneumococcal meningitis outbreak definition [26]: it is unclear whether the criterion of >10 confirmed pneumococcal meningitis cases refers to a single week or overall during the outbreak. We assumed this criterion referred to a single week for the purposes of this analysis. While an alternative interpretation of the criteria of >60% of confirmed meningitis cases caused by *S. pneumoniae* and >10 confirmed cases of pneumococcal meningitis could be considered on a cumulative basis rather than a weekly basis, a challenge remains in defining the time frame for calculating these cumulative measures, especially in districts that may cross the weekly incidence threshold of >5 suspected cases per 100,000 population in non-consecutive weeks, such as in Houndé in 2014. Additionally, an outbreak definition using weekly measures would be most useful to inform real-time outbreak response, including potential reactive vaccination. Finally, during our limited years of observation, we did not detect any pneumococcal meningitis outbreaks in Burkina Faso similar in size to those recently reported in Ghana [8], [25]. Therefore, this analysis is unable to help inform models of pneumococcal meningitis outbreaks in the meningitis belt [27].

This analysis adds to the growing literature regarding pneumococcal meningitis in the context of PCV implementation in West Africa [8], [20], [21], [23], [24], [25], [27], [44], [45], [46], [47], [48], [49], [50], [51], from a country with nationwide population-based surveillance data with routine pneumococcal serotyping [15], [16], [18]. Following the successful introductions of Hib vaccine and MACV, the epidemiology of bacterial meningitis in the meningitis belt is changing and *S. pneumoniae* is increasingly recognized as causing a high proportion of remaining disease [9]. Though we did not identify any large pneumococcal meningitis outbreaks with a weekly incidence of >7 cases or duration >4 weeks in Burkina Faso, we provide data on smaller pneumococcal meningitis clusters to help inform discussions regarding potential prevention and response measures for pneumococcal meningitis in the context of the meningitis belt. We also reaffirmed the predominance of serotype 1 in pneumococcal meningitis clusters and outbreaks [8], [43]. Though we did not retrospectively identify any clear cases where reactive vaccination with PCV could have played a role in outbreak response, continued robust meningitis surveillance [29], real-time specimen tracking [52], high levels of laboratory confirmation [53] and pneumococcal serotyping in Burkina Faso will allow for monitoring and detection of any future pneumococcal meningitis clusters or outbreaks, as well as evaluation of a potential change from a 3 + 0 schedule to a 2 + 1 schedule and how that may impact pneumococcal meningitis dynamics in Burkina Faso.

## CRediT authorship contribution statement

**Heidi M. Soeters:** Formal analysis, Methodology, Writing - original draft. **Dinanibè Kambiré:** Investigation, Validation. **Guetawendé Sawadogo:** Investigation, Validation. **Rasmata Ouédraogo-Traoré:** Project administration, Supervision. **Brice Bicaba:** Investigation, Project administration. **Isaïe Medah:** Funding acquisition. **Lassana Sangaré:** Investigation. **Abdoul-Salam Ouédraogo:** Investigation. **Soumeya Ouangraoua:** Investigation. **Issaka Yaméogo:** Investigation, Validation. **Malika Congo-Ouédraogo:** Investigation. **Absatou Ky Ba:** Investigation. **Flavien Aké:** Project administration, Resources. **Srinivasan Velusamy:** Supervision, Validation. **Lesley McGee:** Supervision, Validation, Writing - review & editing. **Chris Van Beneden:** Funding acquisition, Methodology, Project administration, Supervision, Writing - review & editing. **Cynthia G. Whitney:** Funding acquisition, Methodology, Supervision, Writing - review & editing.

## Declaration of Competing Interest

The authors declare that they have no known competing financial interests or personal relationships that could have appeared to influence the work reported in this paper.

## References

[1] O'Brien K.L., Wolfson L.J., Watt J.P., Henkle E., Deloria-Knoll M., McCall N. (2009). Burden of disease caused by *Streptococcus pneumoniae* in children younger than 5 years: global estimates. Lancet.

[2] Global Burden of Disease Meningitis Collaborators (2018). Global, regional, and national burden of meningitis, 1990–2016: a systematic analysis for the Global Burden of Disease Study 2016. Lancet Neurol.

[3] Global Burden of Disease Collaborative Network (2018). Global Burden of Disease Study 2017 (GBD 2017) Seattle, Washington.

[4] Parent du Chatelet I., Traore Y., Gessner B.D., Antignac A., Naccro B.N., Ouedraogo M.S. (2005). Bacterial Meningitis in Burkina Faso: Surveillance Using Field-Based Polymerase Chain Reaction Testing. Clin Infect Dis.

[5] Yaro S., Lourd M., Traore Y., Njanpop-LaFourcade B.M., Sawadogo A., Sangare L. (2006). Epidemiological and Molecular Characteristics of a Highly Lethal Pneumococcal Meningitis Epidemic in Burkina Faso. Clin Infect Dis.

[6] Traore Y., Tameklo T.A., Njanpop-Lafourcade B.M., Lourd M., Yaro S., Niamba D. (2009). Incidence, seasonality, age distribution, and mortality of pneumococcal meningitis in Burkina Faso and Togo. Clin Infect Dis.

[7] Gessner B.D., Sanou O., Drabo A., Tamekloe T.A., Yaro S., Tall H. (2012). Pneumococcal serotype distribution among meningitis cases from Togo and Burkina Faso during 2007–2009. Vaccine.

[8] Bozio C.H., Abdul-Karim A., Abenyeri J., Abubakari B., Ofosu W., Zoya J. (2018). Continued occurrence of serotype 1 pneumococcal meningitis in two regions located in the meningitis belt in Ghana five years after introduction of 13-valent pneumococcal conjugate vaccine. PLoS ONE.

[9] Soeters H.M., Diallo A.O., Bicaba B.W., Kadade G., Dembele A.Y., Acyl M.A. (2019). Bacterial Meningitis Epidemiology in Five Countries in the Meningitis Belt of Sub-Saharan Africa, 2015–2017. J Infect Dis.

[10] Molesworth A.M., Cuevas L.E., Connor S.J., Morse A.P., Thomson M.C. (2003). Environmental risk and meningitis epidemics in Africa. Emerg Infect Dis.

[11] Sanou I., Bonkoungou I.J.O., Bicaba I., Ouedraogo A., Soudre F., Zeba S. (2014). Hospital-based Sentinel Surveillance of *Haemophilus influenzae* Type b among Children in Burkina Faso, 2004–2012: Impact of Vaccine Introduction. J Med Microb Diagn.

[12] Novak R.T., Kambou J.L., Diomande F.V., Tarbangdo T.F., Ouedraogo-Traore R., Sangare L. (2012). Serogroup A meningococcal conjugate vaccination in Burkina Faso: analysis of national surveillance data. Lancet Infect Dis.

[13] Djingarey M.H., Barry R., Bonkoungou M., Tiendrebeogo S., Sebgo R., Kandolo D. (2012). Effectively introducing a new meningococcal A conjugate vaccine in Africa: the Burkina Faso experience. Vaccine.

[14] World Health Organization. Burkina Faso: WHO and UNICEF estimates of immunization coverage: 2017 revision. 2018.

[15] Soeters H.M., Kambire D., Sawadogo G., Ouedraogo-Traore R., Bicaba B., Medah I. (2019). Impact of 13-Valent Pneumococcal Conjugate Vaccine on Pneumococcal Meningitis, Burkina Faso, 2016–2017. J Infect Dis.

[16] Kambire D., Soeters H.M., Ouedraogo-Traore R., Medah I., Sangare L., Yameogo I. (2018). Early impact of 13-valent pneumococcal conjugate vaccine on pneumococcal meningitis-Burkina Faso, 2014–2015. J Infect.

[17] Diallo A.O., Soeters H.M., Yameogo I., Sawadogo G., Aké F., Lingani C. (2017). Bacterial meningitis epidemiology and return of *Neisseria meningitidis* serogroup A cases in Burkina Faso in the five years following MenAfriVac mass vaccination campaign. PLoS ONE.

[18] Kambire D., Soeters H.M., Ouedraogo-Traore R., Medah I., Sangare L., Yameogo I. (2016). Nationwide Trends in Bacterial Meningitis before the Introduction of 13-Valent Pneumococcal Conjugate Vaccine-Burkina Faso, 2011–2013. PLoS ONE.

[19] Whitney C.G. (2018). Examining Duration of Protection: Should a Booster Dose Be Part of All Infant Pneumococcal Conjugate Vaccine Programs?. Clin Infect Dis.

[20] Tagbo B.N., Bancroft R.E., Fajolu I., Abdulkadir M.B., Bashir M.F., Okunola O.P. (2019). Pediatric Bacterial Meningitis Surveillance in Nigeria From 2010 to 2016, Prior to and During the Phased Introduction of the 10-Valent Pneumococcal Conjugate Vaccine. Clin Infect Dis.

[21] Sonko M.A., Dube F.S., Okoi C.B., Diop A., Thiongane A., Senghore M. (2019). Changes in the Molecular Epidemiology of Pediatric Bacterial Meningitis in Senegal After Pneumococcal Conjugate Vaccine Introduction. Clin Infect Dis.

[22] Mwenda J.M., Soda E., Weldegebriel G., Katsande R., Biey J.N., Traore T. (2019). Pediatric Bacterial Meningitis Surveillance in the World Health Organization African Region Using the Invasive Bacterial Vaccine-Preventable Disease Surveillance Network, 2011–2016. Clin Infect Dis.

[23] Boni-Cisse C., Jarju S., Bancroft R.E., Lepri N.A., Kone H., Kofi N. (2019). Etiology of Bacterial Meningitis Among Children <5 Years Old in Cote d'Ivoire: Findings of Hospital-based Surveillance Before and After Pneumococcal Conjugate Vaccine Introduction. Clin Infect Dis.

[24] Aku F.Y., Lessa F.C., Asiedu-Bekoe F., Balagumyetime P., Ofosu W., Farrar J. (2017). Meningitis Outbreak Caused by Vaccine-Preventable Bacterial Pathogens - Northern Ghana, 2016. MMWR.

[25] Kwambana-Adams B.A., Asiedu-Bekoe F., Sarkodie B., Afreh O.K., Kuma G.K., Owusu-Okyere G. (2016). An outbreak of pneumococcal meningitis among older children (≥5 years) and adults after the implementation of an infant vaccination programme with the 13-valent pneumococcal conjugate vaccine in Ghana. BMC Infect Dis.

[26] Pneumococcal meningitis outbreaks in sub-Saharan Africa (2016). Wkly Epidemiol Rec.

[27] Cooper L.V., Stuart J.M., Okot C., Asiedu-Bekoe F., Afreh O.K., Fernandez K. (2019). Reactive vaccination as a control strategy for pneumococcal meningitis outbreaks in the African meningitis belt: Analysis of outbreak data from Ghana. Vaccine.

[28] World Health Organization. Technical Guidelines for Integrated Disease Surveillance and Response in the African Region, 2nd Edition. Brazzaville; 2010.

[29] Patel J.C., Soeters H.M., Diallo A.O., Bicaba B.W., Kadade G., Dembele A.Y. (2019). MenAfriNet: A Network Supporting Case-Based Meningitis Surveillance and Vaccine Evaluation in the Meningitis Belt of Africa. J Infect Dis.

[30] Standard WHO-AFRO. (2009). operating procedures for enhanced meningitis surveillance in Africa. World Health Organization Regional.

[31] Carvalho Mda G., Tondella M.L., McCaustland K., Weidlich L., McGee L., Mayer L.W. (2007). Evaluation and improvement of real-time PCR assays targeting *lytA*, *ply*, and *psaA* genes for detection of pneumococcal DNA. J Clin Micro.

[32] Pimenta F.C., Roundtree A., Soysal A., Bakir M., du Plessis M., Wolter N. (2013). Sequential triplex real-time PCR assay for detecting 21 pneumococcal capsular serotypes that account for a high global disease burden. J Clin Micro.

[33] Burkina Faso Ministère de la Santé. Annuaire Statistique 2017. Ouagadougou. 2018.

[34] World Health Organization (2019). International Coordinating Group (ICG) on Vaccine Provision.

[35] World Health Organization (2019). Reform of the International Coordinating Group for Vaccine Provision: a new framework for coordination, accountability and transparency. Wkly Epidemiol Rec.

[36] Fernandez K., Lingani C., Aderinola O.M., Goumbi K., Bicaba B., Edea Z.A. (2019). Meningococcal Meningitis Outbreaks in the African Meningitis Belt After Meningococcal Serogroup A Conjugate Vaccine Introduction, 2011–2017. J Infect Dis.

[37] Klugman K.P., Madhi S.A., Adegbola R.A., Cutts F., Greenwood B., Hausdorff W.P. (2011). Timing of serotype 1 pneumococcal disease suggests the need for evaluation of a booster dose. Vaccine.

[38] Pneumococcal conjugate vaccines in infants and children under 5 years of age: WHO position paper – February 2019. Weekly Epidemiological Record. 2019;8:85–104.

[39] Pneumococcal vaccines WHO position paper – 2012. Weekly Epidemiological Record. 2012;87:129–44. 24340399

[40] Heinsbroek E., Tafatatha T., Phiri A., Swarthout T.D., Alaerts M., Crampin A.C. (2018). Pneumococcal carriage in households in Karonga District, Malawi, before and after introduction of 13-valent pneumococcal conjugate vaccination. Vaccine.

[41] Hill P.C., Townend J., Antonio M., Akisanya B., Ebruke C., Lahai G. (2010). Transmission of Streptococcus pneumoniae in rural Gambian villages: a longitudinal study. Clin Infect Dis.

[42] Tigoi C.C., Gatakaa H., Karani A., Mugo D., Kungu S., Wanjiru E. (2012). Rates of acquisition of pneumococcal colonization and transmission probabilities, by serotype, among newborn infants in Kilifi District. Kenya Clin Infect Dis.

[43] Coldiron M.E., Toure O., Frank T., Bouygues N., Grais R.F. (2018). Outbreak of Pneumococcal Meningitis, Paoua Subprefecture, Central African Republic, 2016–2017. Emerg Infect Dis.

[44] Mackenzie G.A., Hill P.C., Jeffries D.J., Hossain I., Uchendu U., Ameh D. (2016). Effect of the introduction of pneumococcal conjugate vaccination on invasive pneumococcal disease in The Gambia: a population-based surveillance study. Lancet Infect Dis.

[45] Kourna Hama M., Khan D., Laouali B., Okoi C., Yam A., Haladou M. (2019). Pediatric Bacterial Meningitis Surveillance in Niger: Increased Importance of *Neisseria meningitidis* Serogroup C, and a Decrease in *Streptococcus pneumoniae* Following 13-Valent Pneumococcal Conjugate Vaccine Introduction. Clin Infect Dis.

[46] Sanneh B., Okoi C., Grey-Johnson M., Bah-Camara H., Kunta Fofana B., Baldeh I. (2019). Declining Trends of Pneumococcal Meningitis in Gambian Children After the Introduction of Pneumococcal Conjugate Vaccines. Clin Infect Dis.

[47] Agossou J., Ebruke C., Noudamadjo A., Adedemy J.D., Denon E.Y., Bankole H.S. (2019). Declines in Pediatric Bacterial Meningitis in the Republic of Benin Following Introduction of Pneumococcal Conjugate Vaccine: Epidemiological and Etiological Findings, 2011–2016. Clin Infect Dis.

[48] Darboe S., Okomo U., Muhammad A.K., Ceesay B., Jallow M., Usuf E. (2019). Community-acquired Invasive Bacterial Disease in Urban Gambia, 2005–2015: A Hospital-based Surveillance. Clin Infect Dis.

[49] Faye P.M., Sonko M.A., Diop A., Thiongane A., Ba I.D., Spiller M. (2019). Impact of 13-Valent Pneumococcal Conjugate Vaccine on Meningitis and Pneumonia Hospitalizations in Children aged <5 Years in Senegal, 2010–2016. Clin Infect Dis.

[50] Tsolenyanu E., Bancroft R.E., Sesay A.K., Senghore M., Fiawoo M., Akolly D. (2019). Etiology of Pediatric Bacterial Meningitis Pre- and Post-PCV13 Introduction Among Children Under 5 Years Old in Lome. Togo Clin Infect Dis.

[51] Renner L.A., Usuf E., Mohammed N.I., Ansong D., Dankwah T., Kusah J.T. (2019). Hospital-based Surveillance for Pediatric Bacterial Meningitis in the Era of the 13-Valent Pneumococcal Conjugate Vaccine in Ghana. Clin Infect Dis.

[52] Diallo A.O., Kiemtore T., Bicaba B.W., Medah I., Tarbangdo T.F., Sanou S. (2019). Development and Implementation of a Cloud-Based Meningitis Surveillance and Specimen Tracking System in Burkina Faso, 2018. J Infect Dis.

[53] Mbaeyi S.A., Lingani C., Diallo A.O., Bicaba B., Ouedraogo-Traore R., Acyl M. (2019). Improving Case-Based Meningitis Surveillance in 5 Countries in the Meningitis Belt of Sub-Saharan Africa, 2015–2017. J Infect Dis.

