# Supplementary material for: Evaluation of pneumococcal meningitis clusters in Burkina Faso and implications for potential reactive vaccination
Source: Vaccine. 2020 Jul 31;38(35):5726–33. doi: 10.1016/j.vaccine.2020.06.002 (PMC7388202; doi:10.1016/j.vaccine.2020.06.002)

**SUPPLEMENTAL MATERIAL**

**Supplemental Table. *S. pneumoniae* diagnostic test results among confirmed^a^ pneumococcal meningitis cases, Burkina Faso, 2011–2017**

| **Diagnostic testing method** | **No. positive for *S. pneumoniae* using method(s) (%)** | |
| --- | --- | --- |
| Latex agglutination only | 402 | (11.5) |
| Culture only | 35 | (1.0) |
| PCR only | 2,251 | (64.4) |
| Latex agglutination and culture only | 27 | (0.8) |
| Latex agglutination and PCR only | 555 | (15.9) |
| Culture and PCR | 117 | (3.3) |
| Latex agglutination, culture and PCR | 111 | (3.2) |
|  | **No. positive / No. of confirmed^a^ cases tested using method (%)** | |
| Latex agglutination | 1,095/1,301 | (84.2) |
| Culture | 290/809 | (35.9) |
| PCR | 3,034/3,115 | (97.4) |
| **Total positive for *S. pneumoniae*** | **3498** |  |

Abbreviations : PCR, polymerase chain reaction.

^a^ *S. pneumoniae* isolated from cerebrospinal fluid (CSF) by culture or detected in CSF by PCR or latex agglutination. Not all specimens were tested via all three methods.

Clusters of ≥5 reported pneumococcal meningitis cases in a district in 1 week, Burkina Faso, 2011–2017

[Dafra 2011, Epidemiologic week 6 3](#_Toc41748335)

[Houndé 2011, Epidemiologic week 8 4](#_Toc41748336)

[Karangasso Vigué 2011, Epidemiologic week 8 5](#_Toc41748337)

[Kaya 2011, Epidemiologic weeks 10–11 6](#_Toc41748338)

[Ouahigouya 2011, Epidemiologic week 9 7](#_Toc41748339)

[Pama 2011, Epidemiologic week 8 8](#_Toc41748340)

[Saponé 2011, Epidemiologic week 3 9](#_Toc41748341)

[Tenkodogo 2011, Epidemiologic week 7 10](#_Toc41748342)

[Dori 2012, Epidemiologic week 10 11](#_Toc41748343)

[Koudougou 2012, Epidemiologic weeks 14–16 12](#_Toc41748344)

[Ouahigouya 2012, Epidemiologic week 15 13](#_Toc41748345)

[Boromo 2013, Epidemiologic week 10 14](#_Toc41748346)

[Houndé 2014, Epidemiologic weeks 2, 3, 5 15](#_Toc41748347)

[Kaya 2017, Epidemiologic week 7 16](#_Toc41748348)

[Titao 2017, Epidemiologic week 4 17](#_Toc41748349)

*Abbreviations:*

*Hi Haemophilus influenzae*

*Nm Neisseria meningitidis*

*NT Nontypeable*

*Sp Streptococcus pneumoniae*

*ST Serotype*

*Notes:*

- *“No serotype” means serotyping was not able to be performed for that case*

# Dafra 2011, Epidemiologic week 6


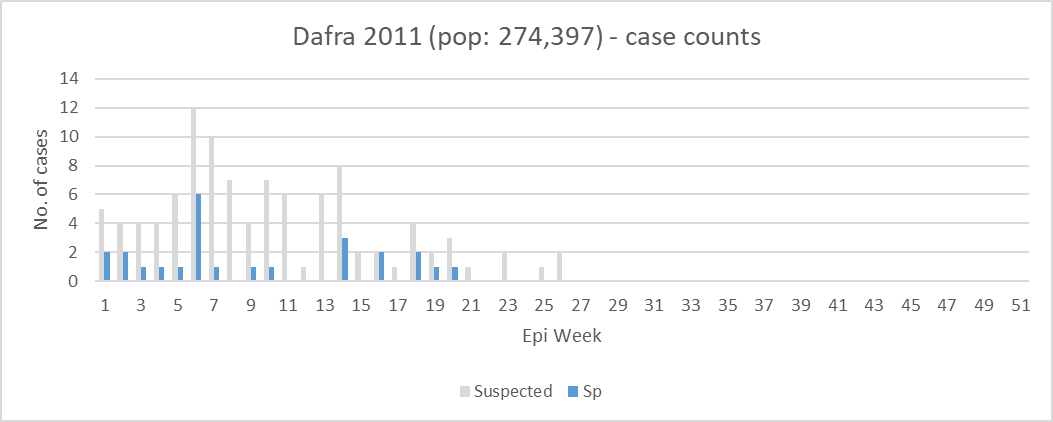

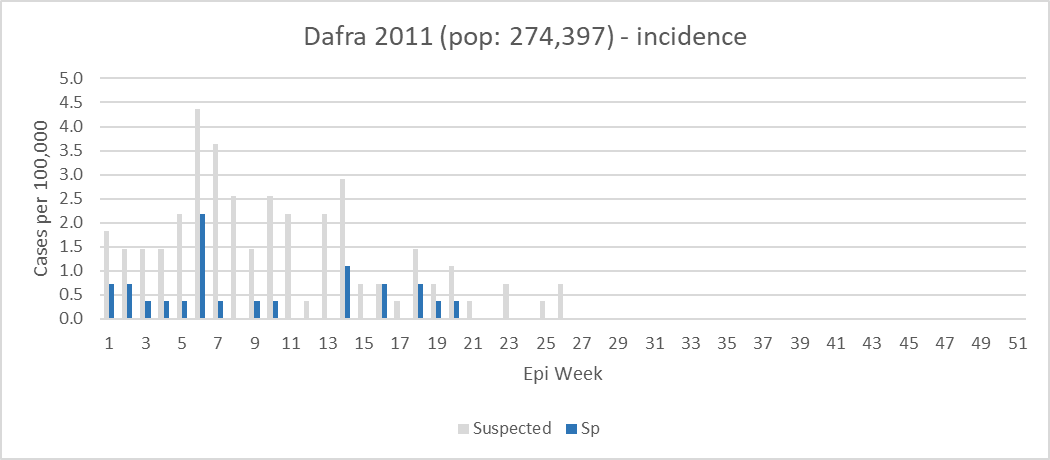

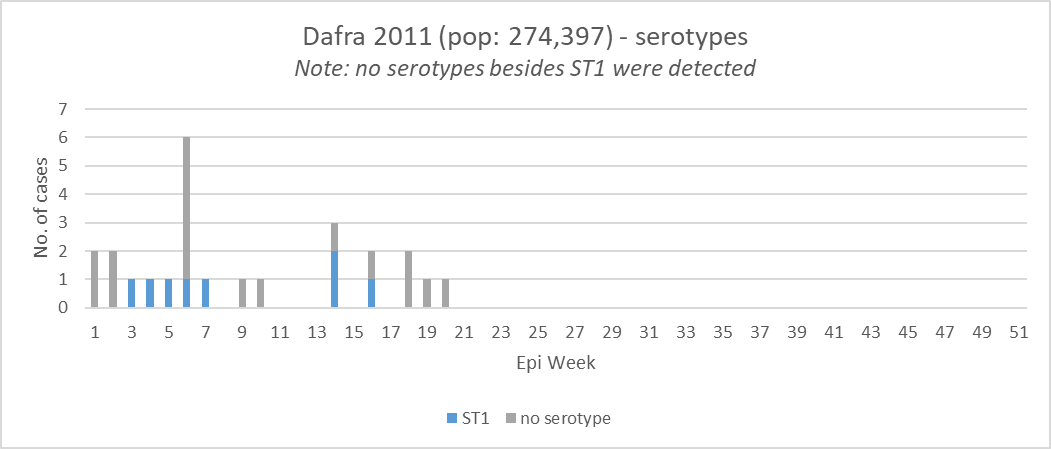

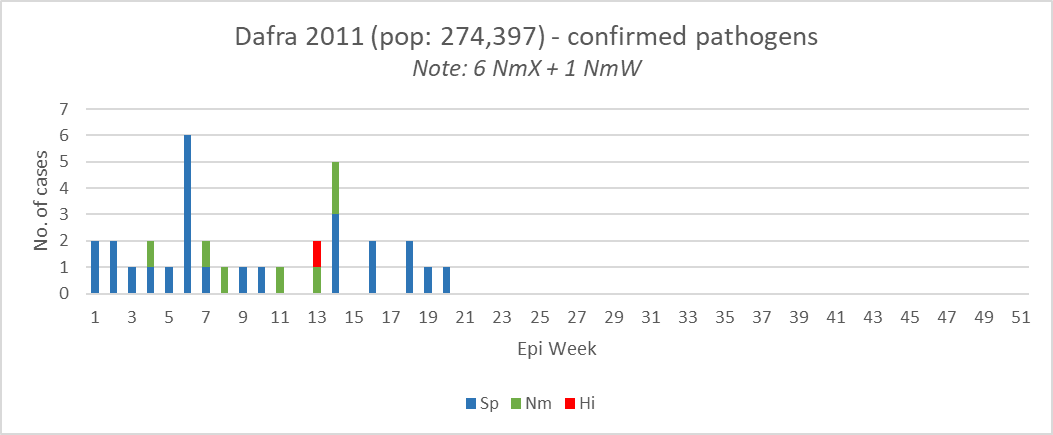


# Houndé 2011, Epidemiologic week 8


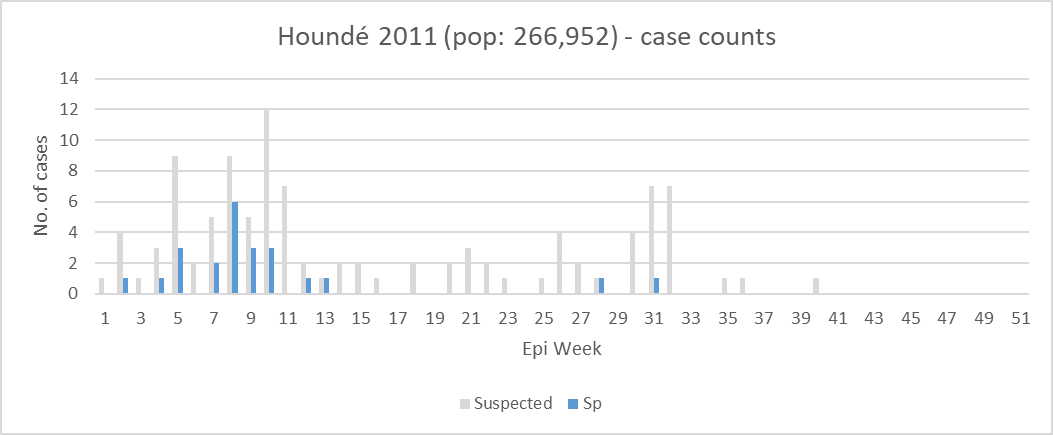

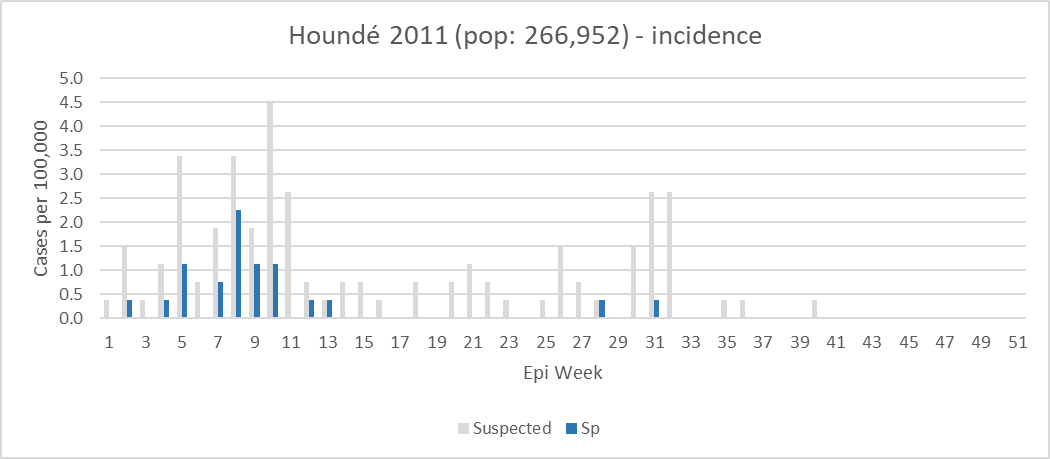

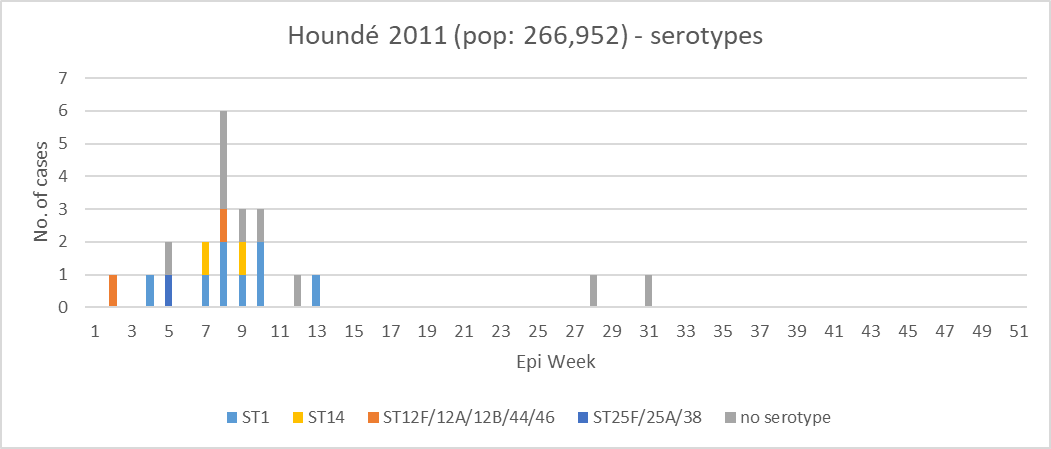

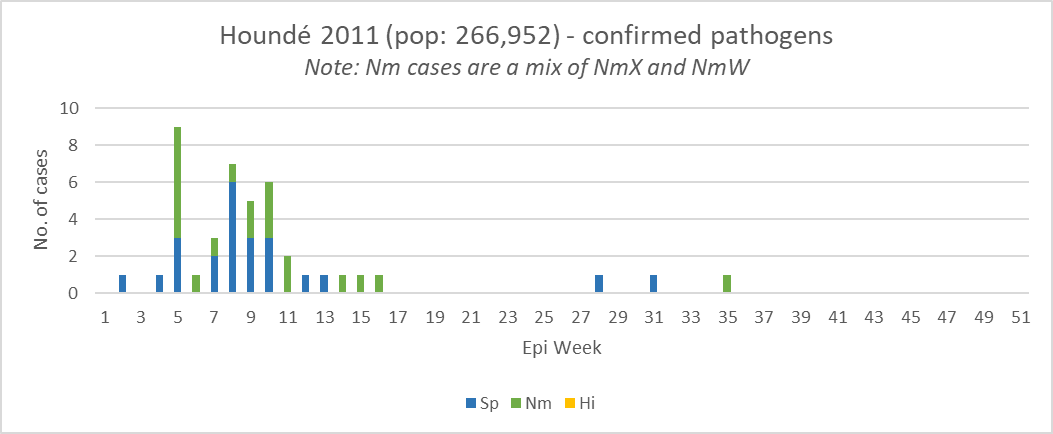


# Karangasso Vigué 2011, Epidemiologic week 8


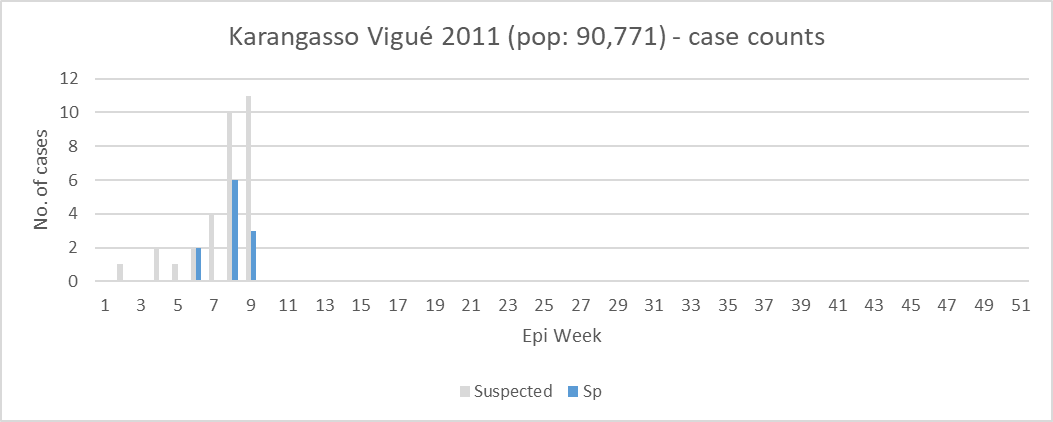

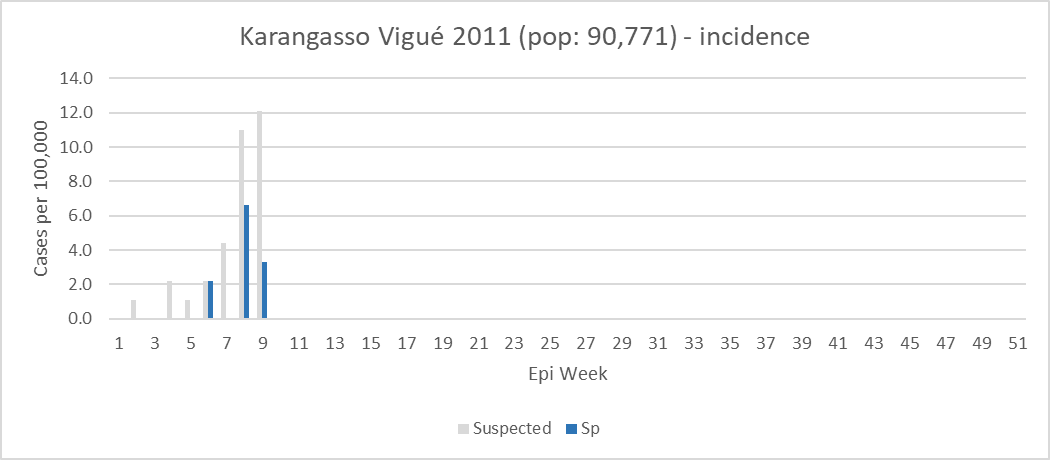

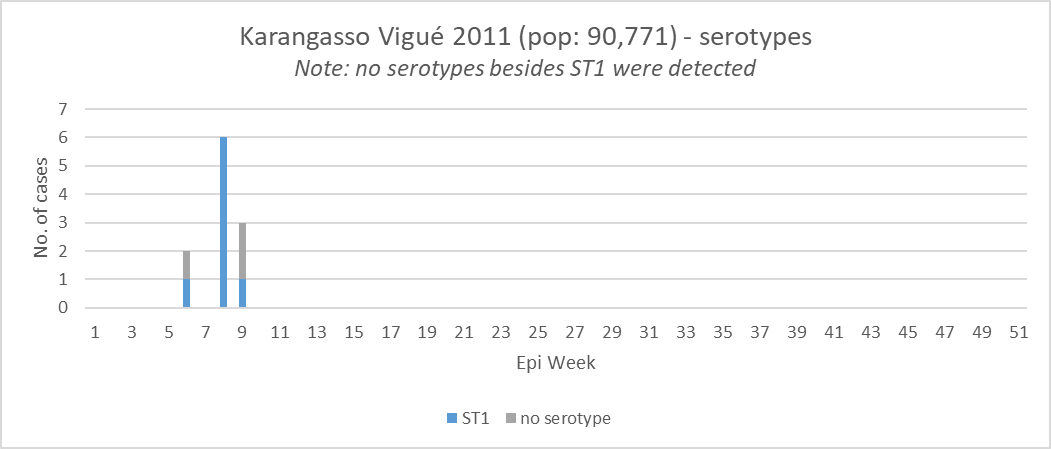

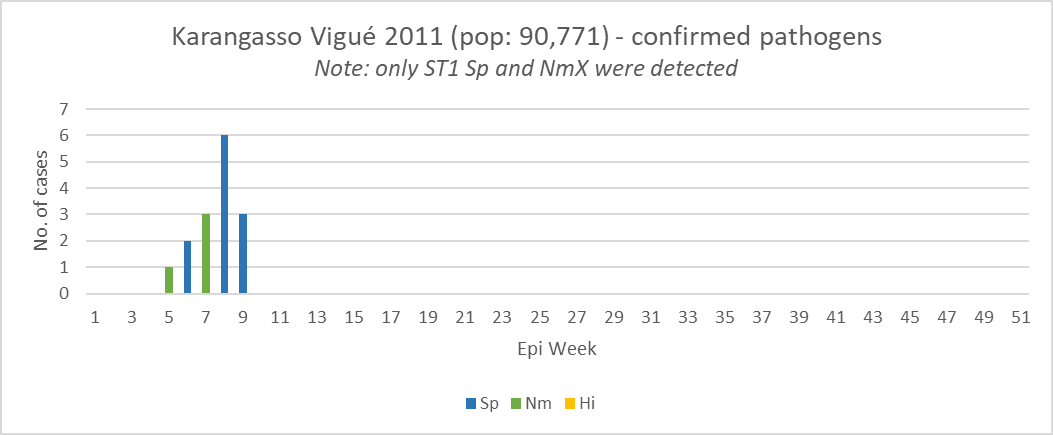


# Kaya 2011, Epidemiologic weeks 10–11


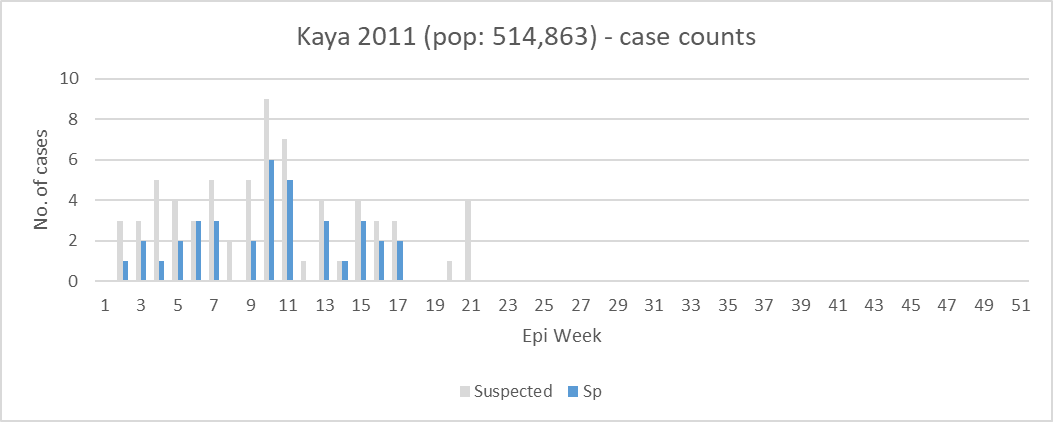

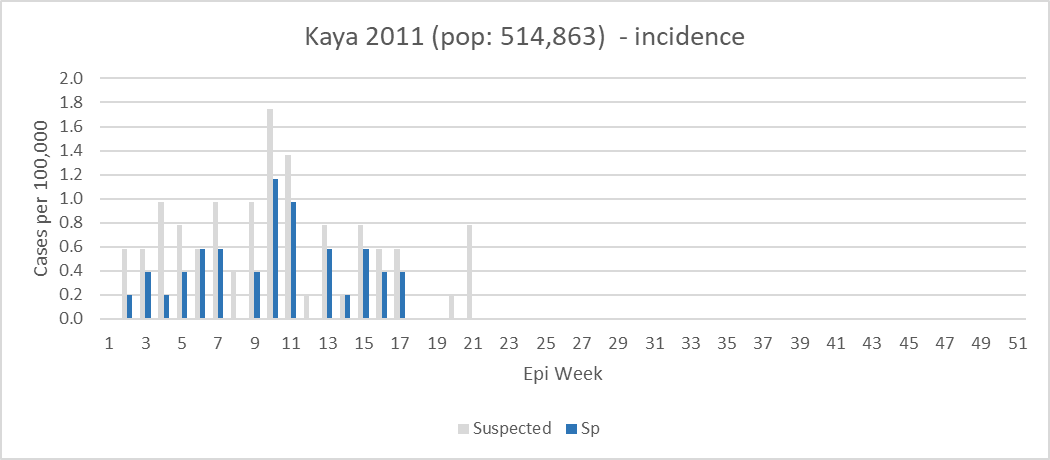

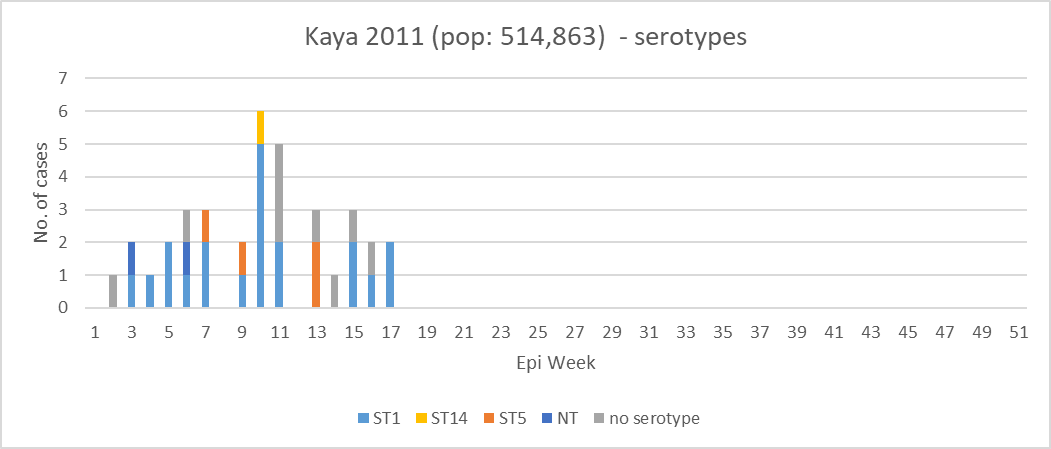

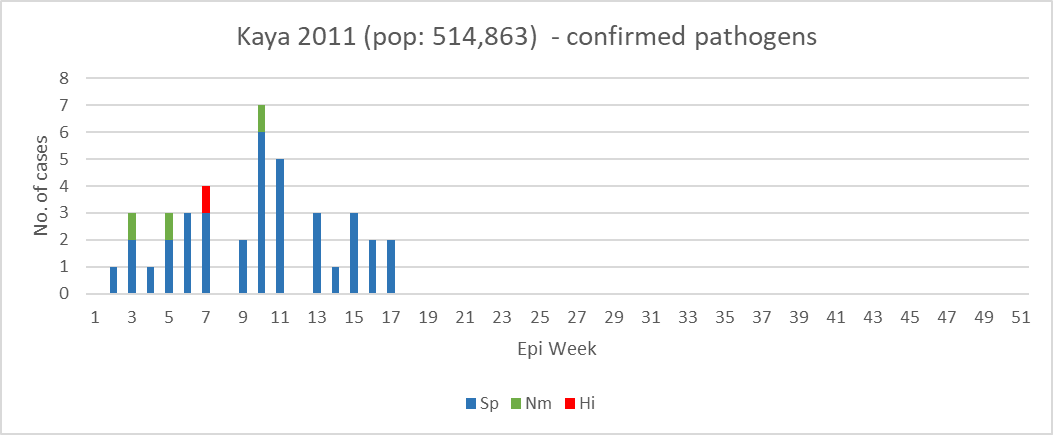


# Ouahigouya 2011, Epidemiologic week 9


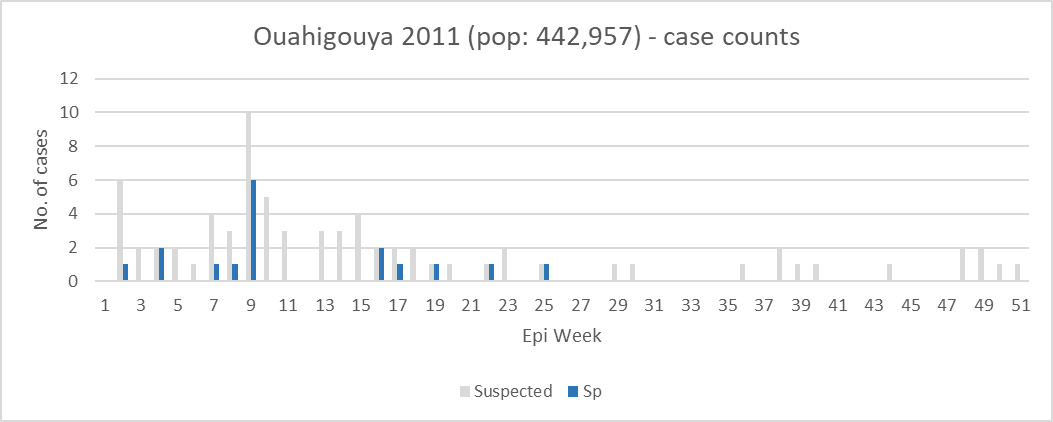

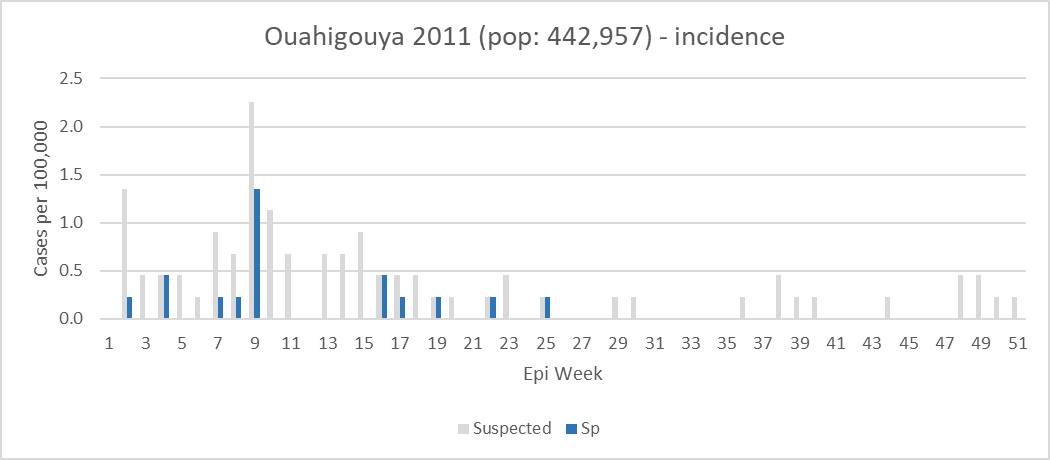

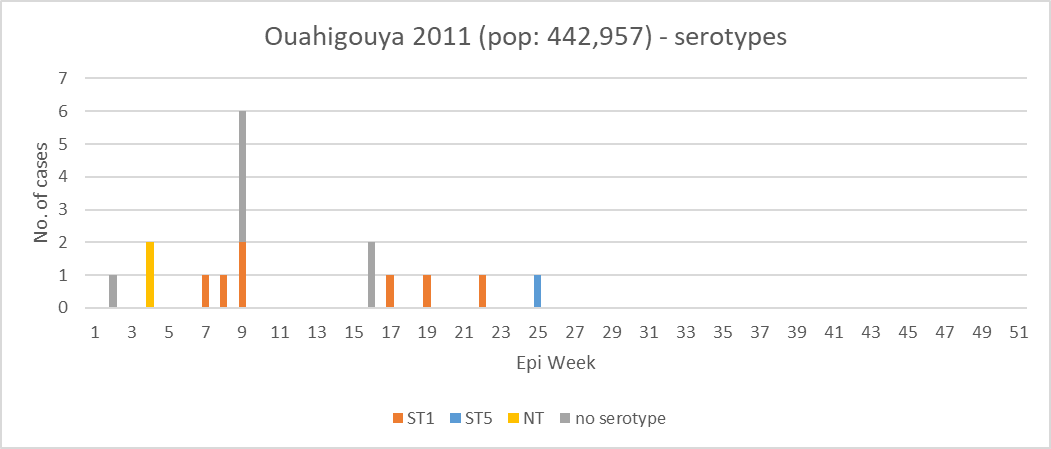

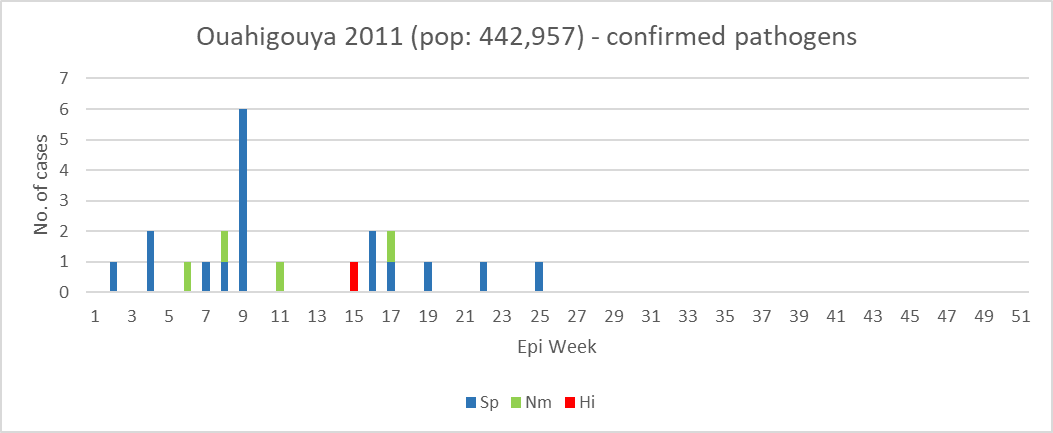


# Pama 2011, Epidemiologic week 8


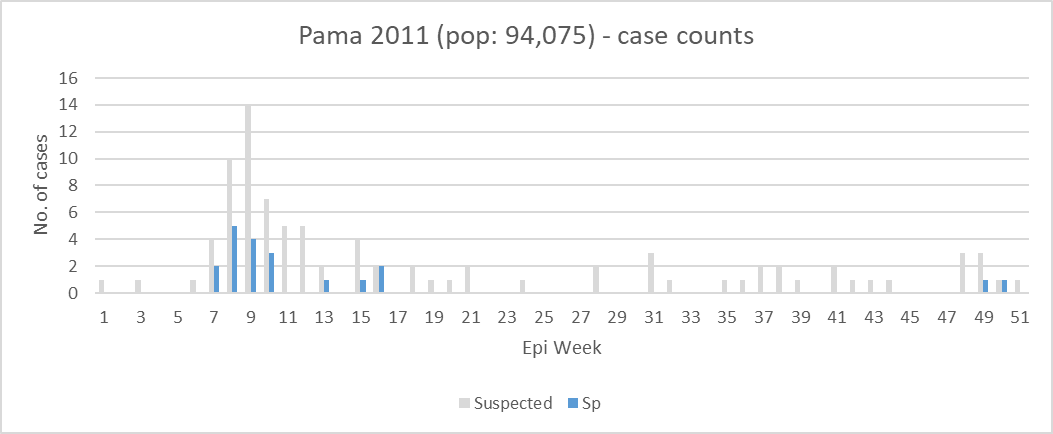

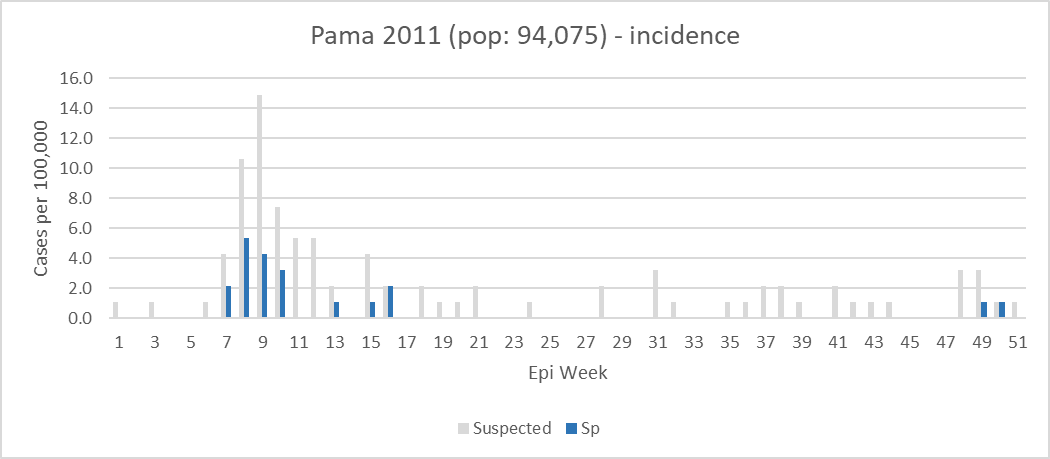

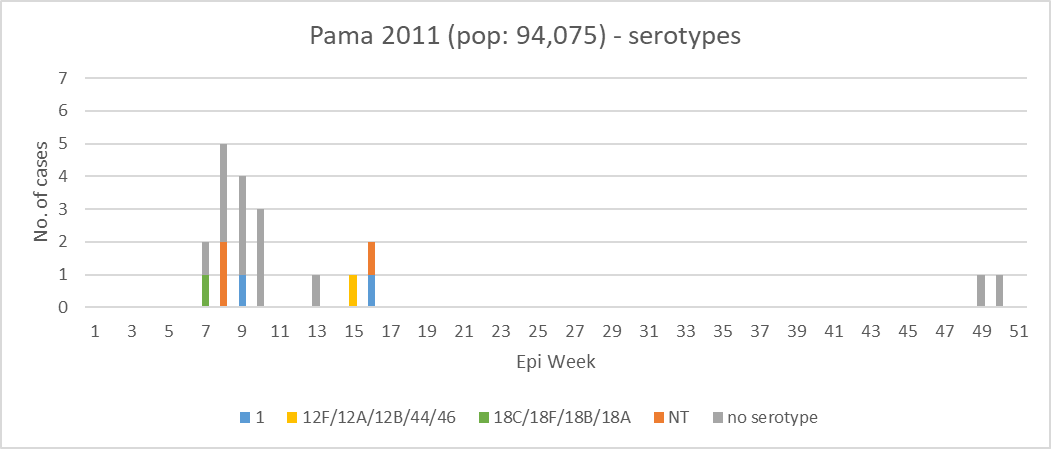

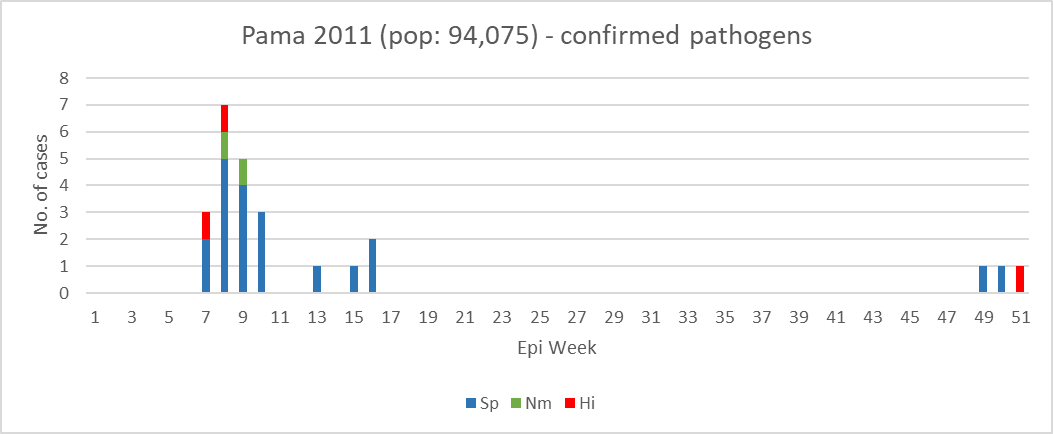


# Saponé 2011, Epidemiologic week 3


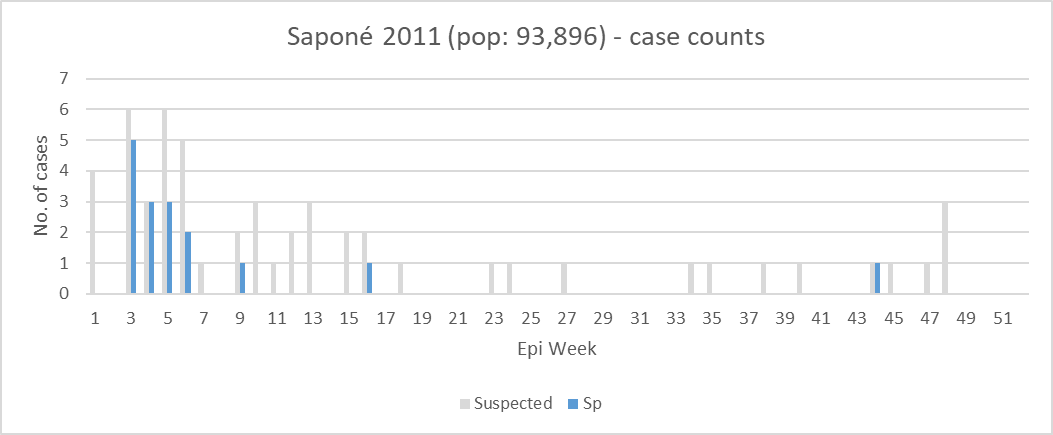

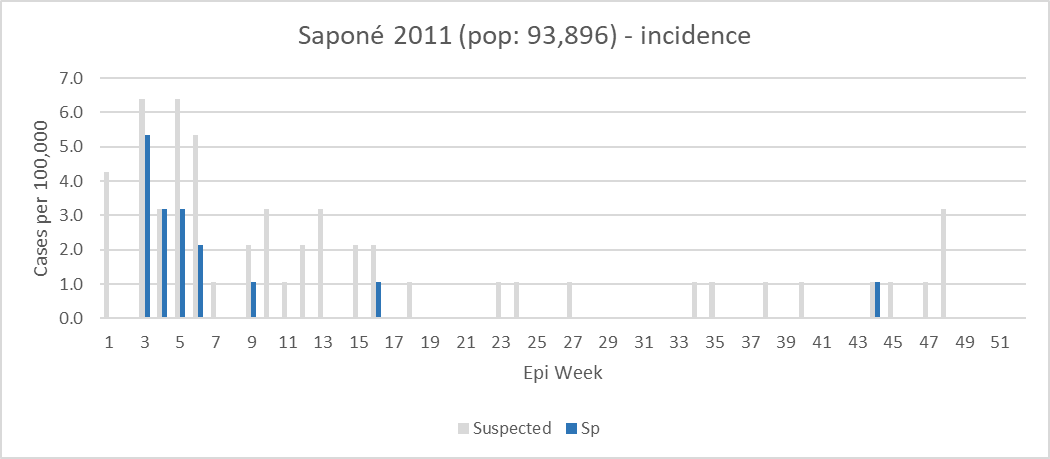

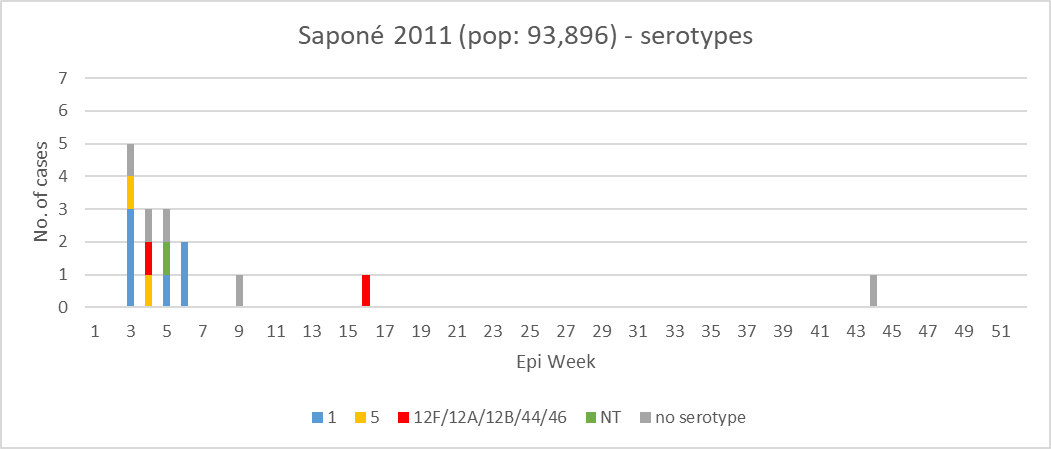

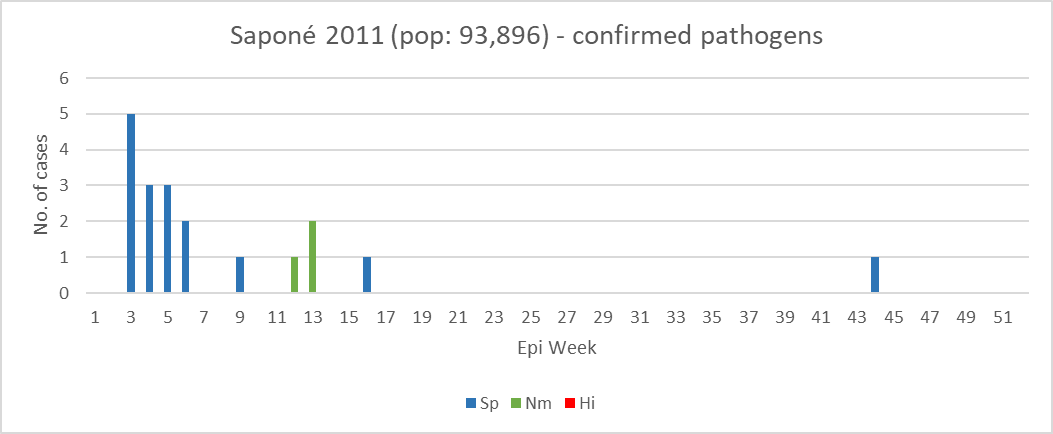


# Tenkodogo 2011, Epidemiologic week 7


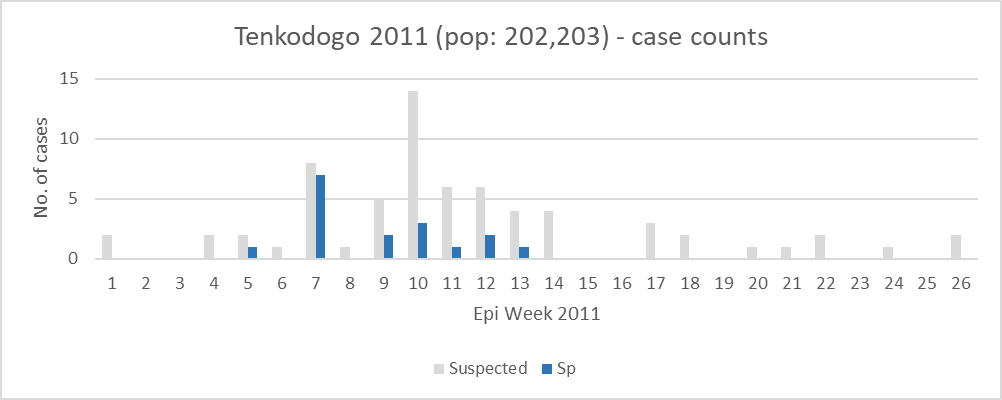

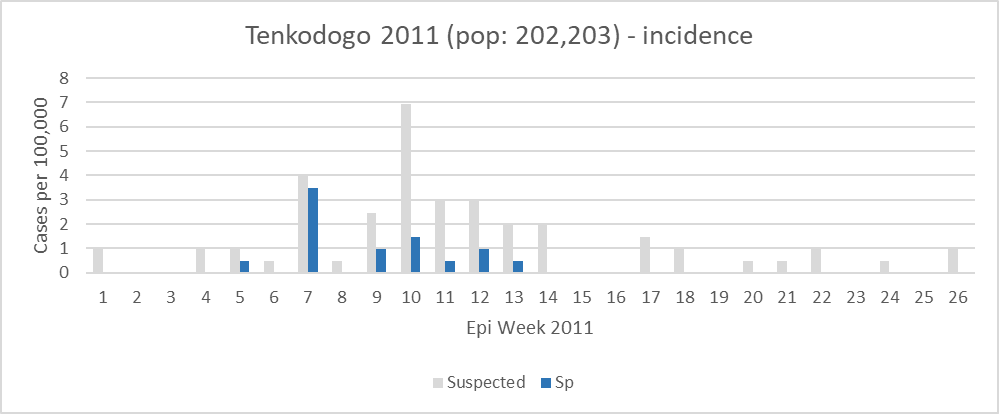

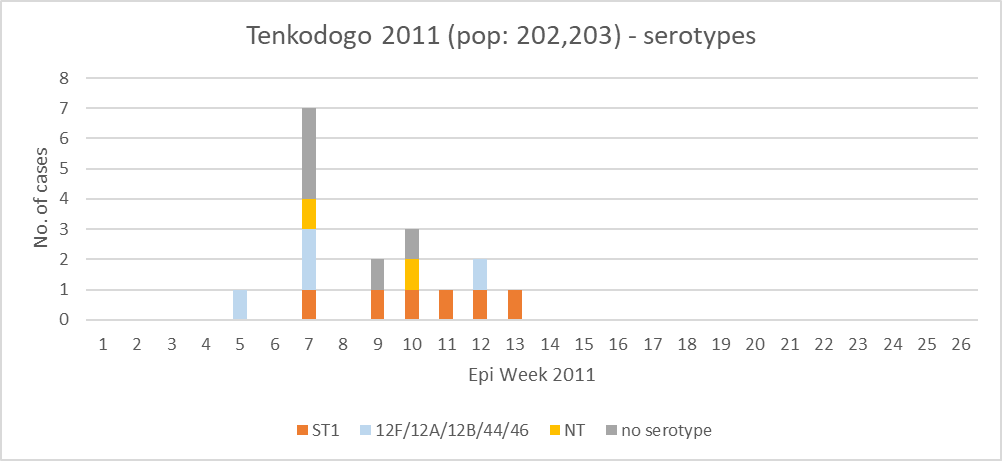

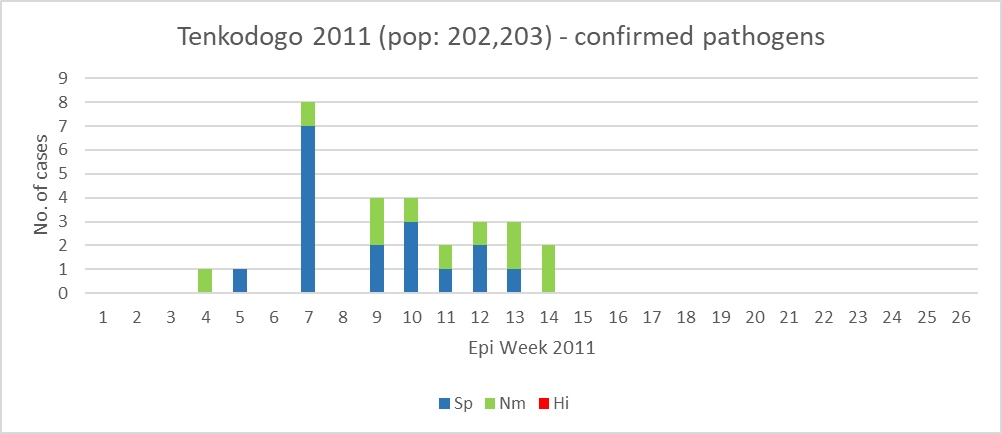


# Dori 2012, Epidemiologic week 10


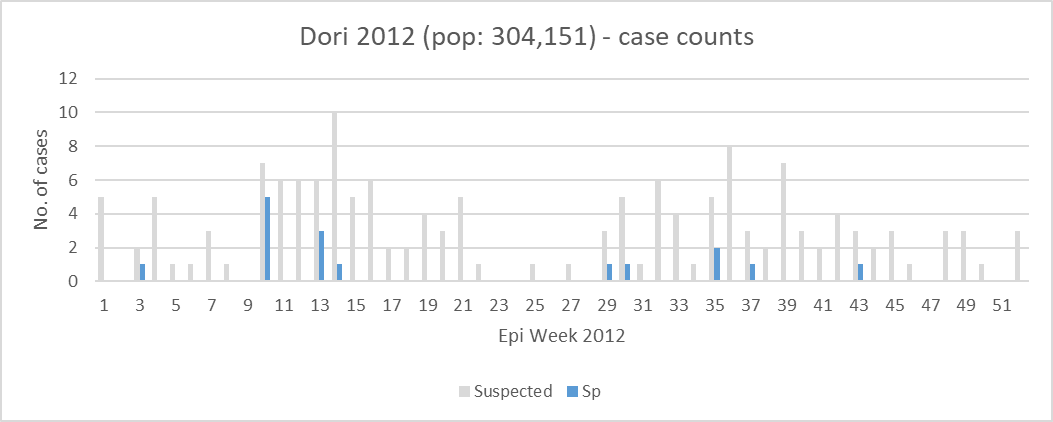

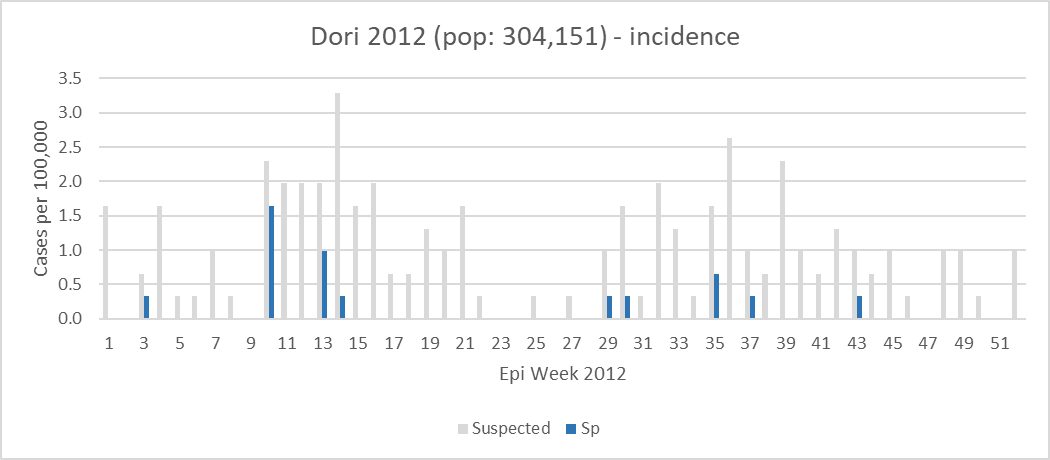

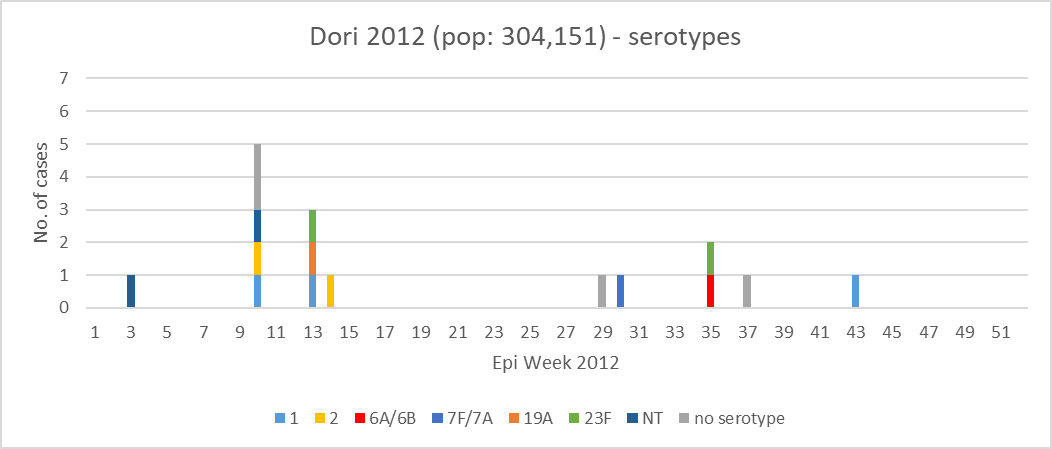

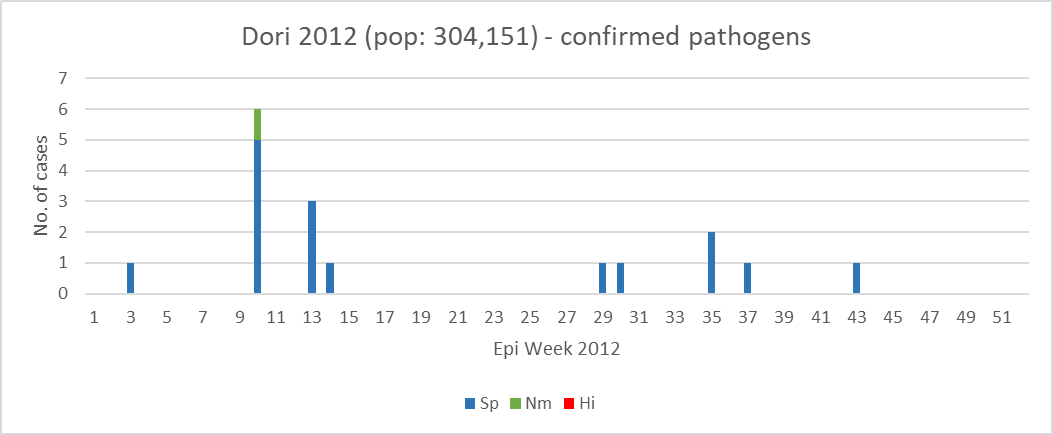


# Koudougou 2012, Epidemiologic weeks 14–16


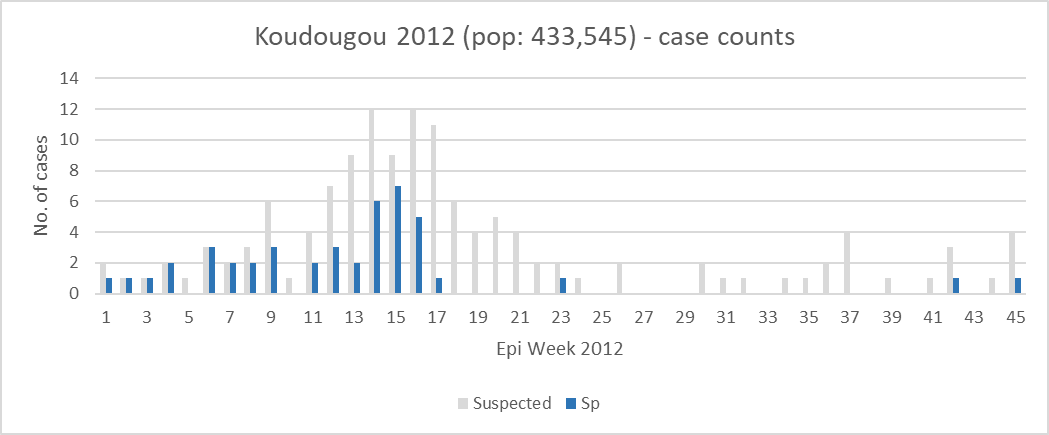

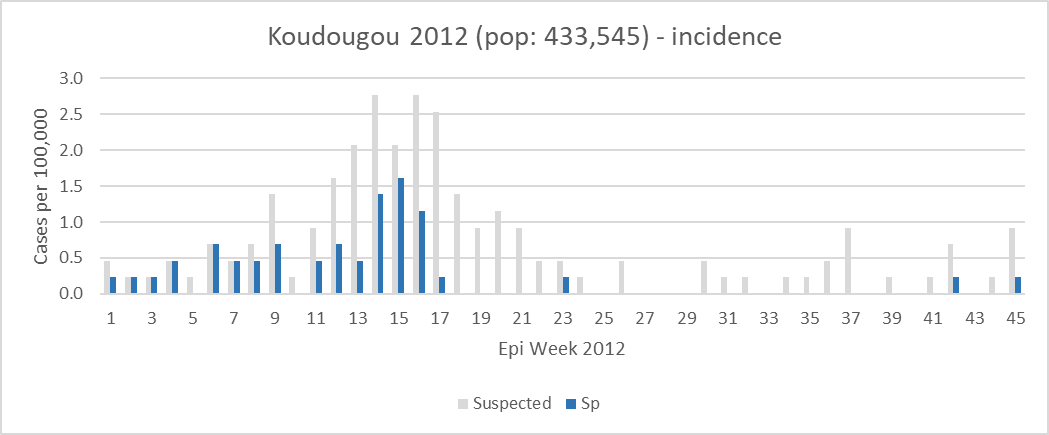

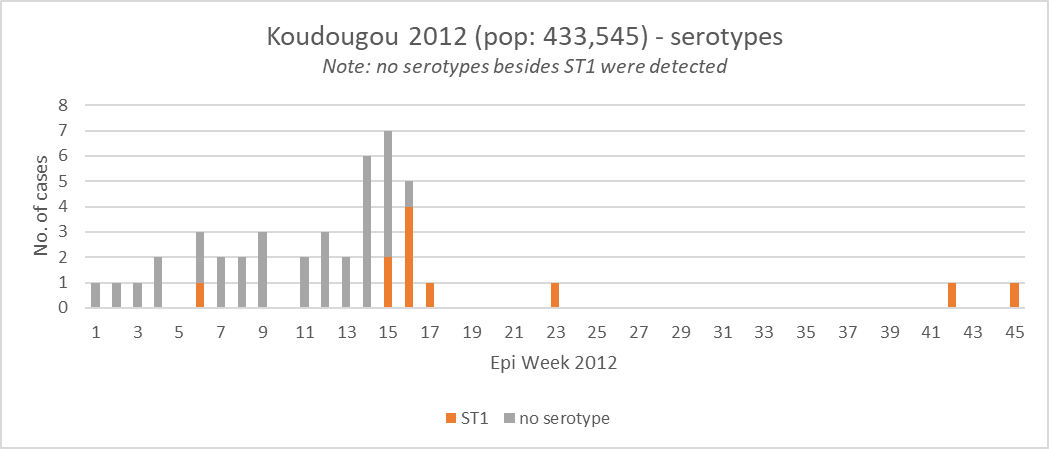

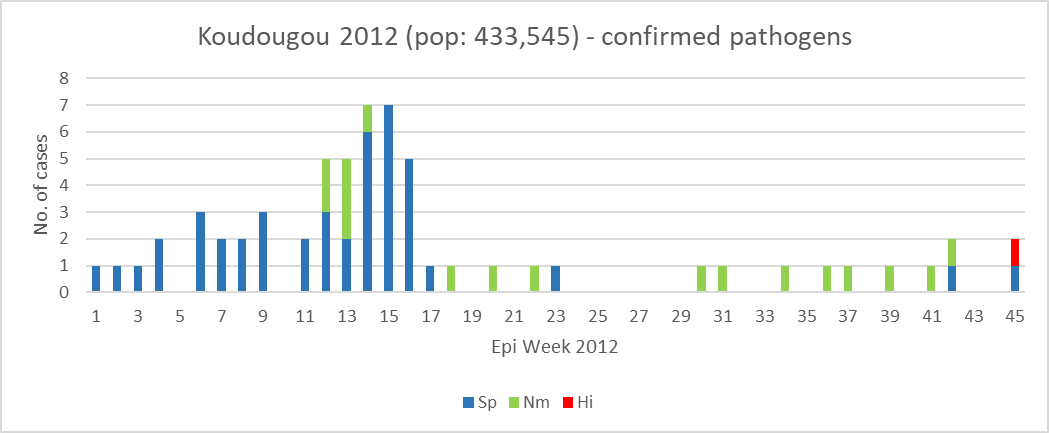


# Ouahigouya 2012, Epidemiologic week 15


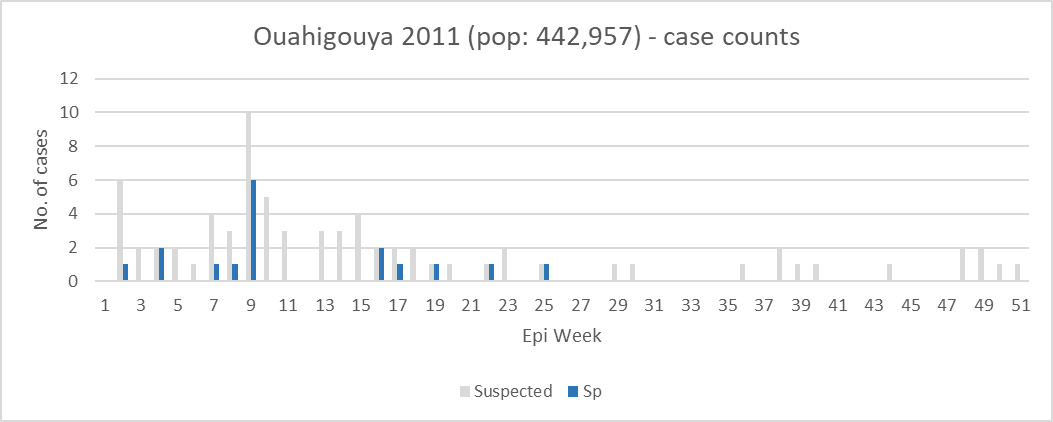

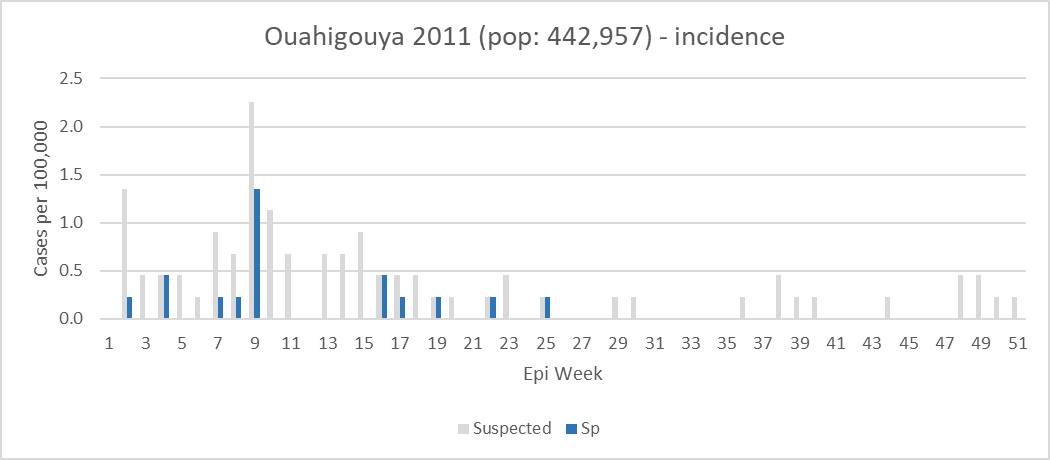

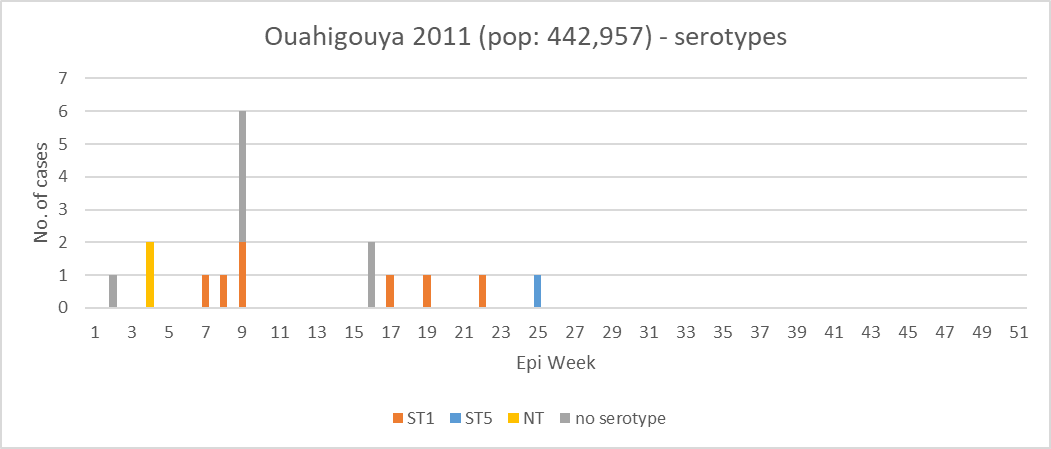

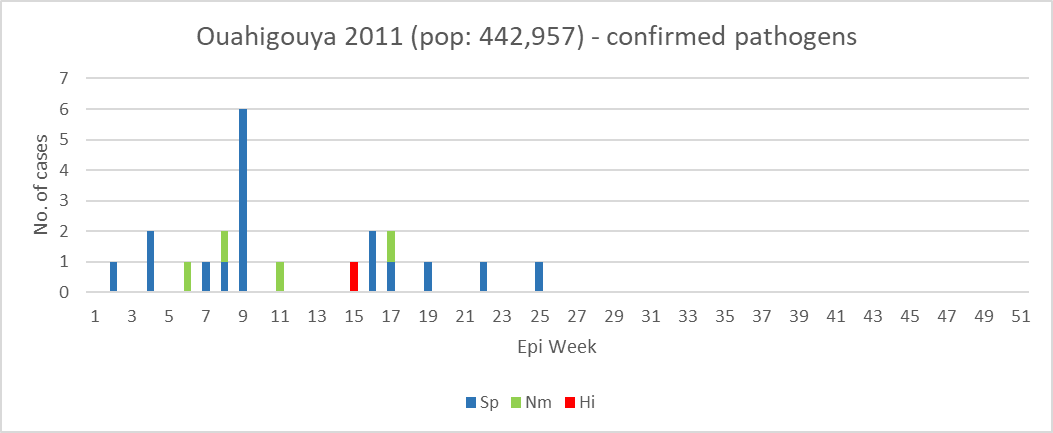


# Boromo 2013, Epidemiologic week 10


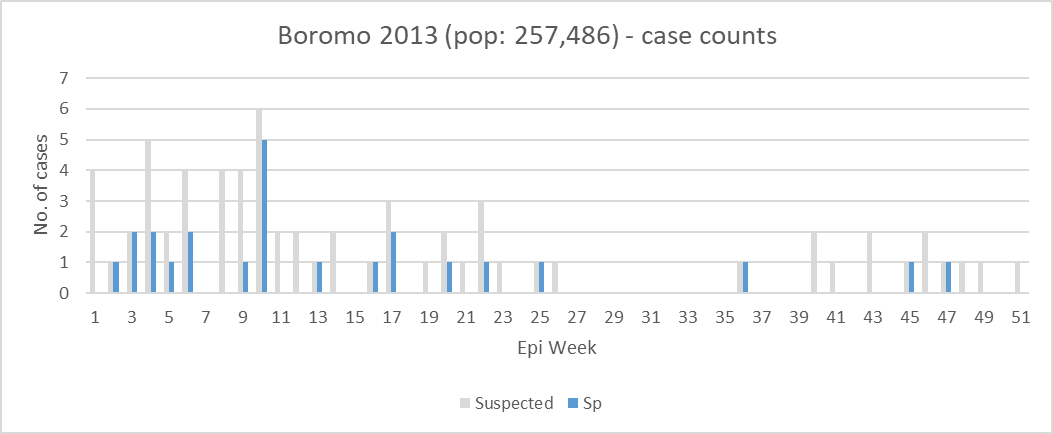

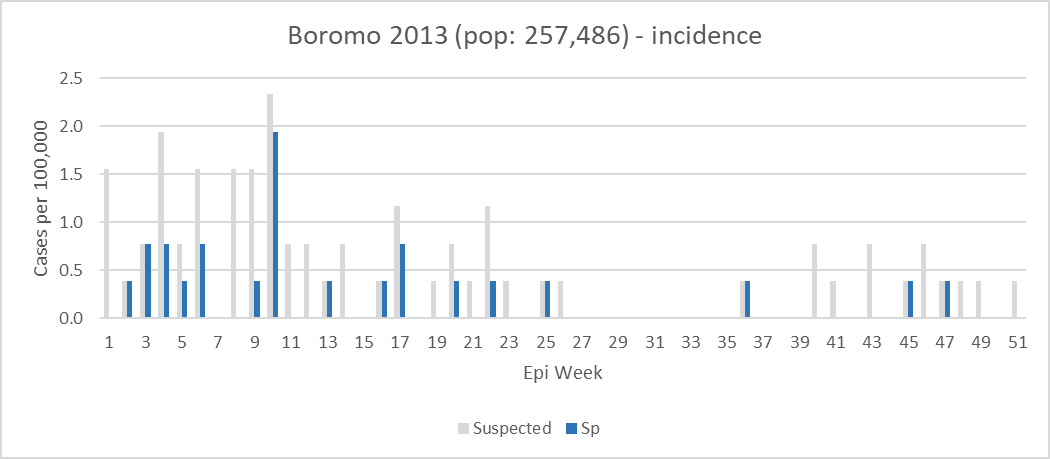

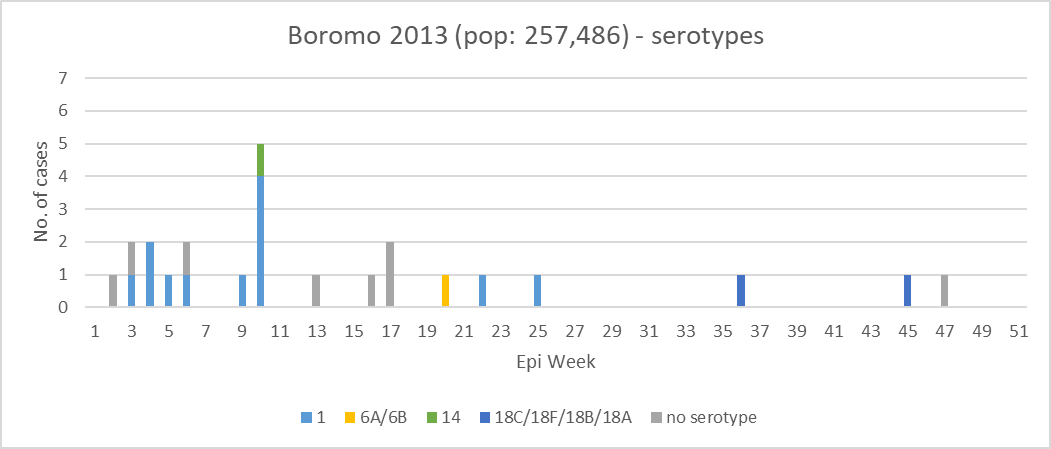

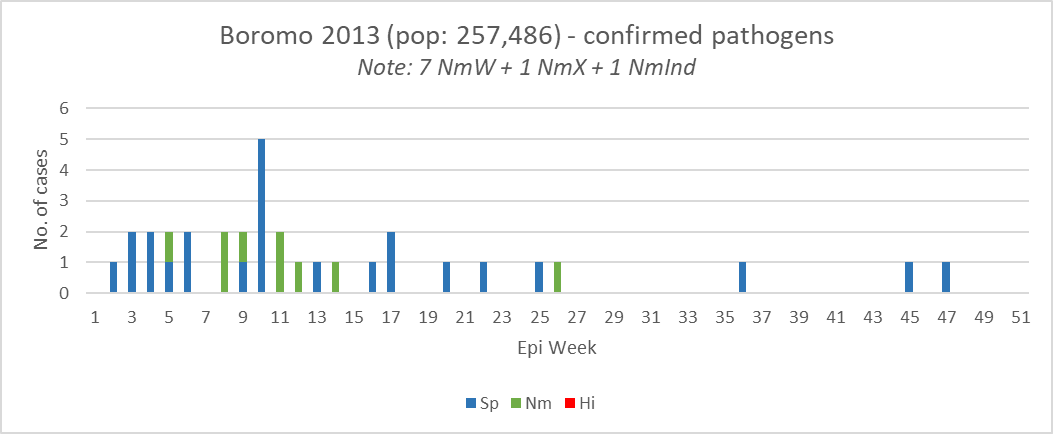


# Houndé 2014, Epidemiologic weeks 2, 3, 5


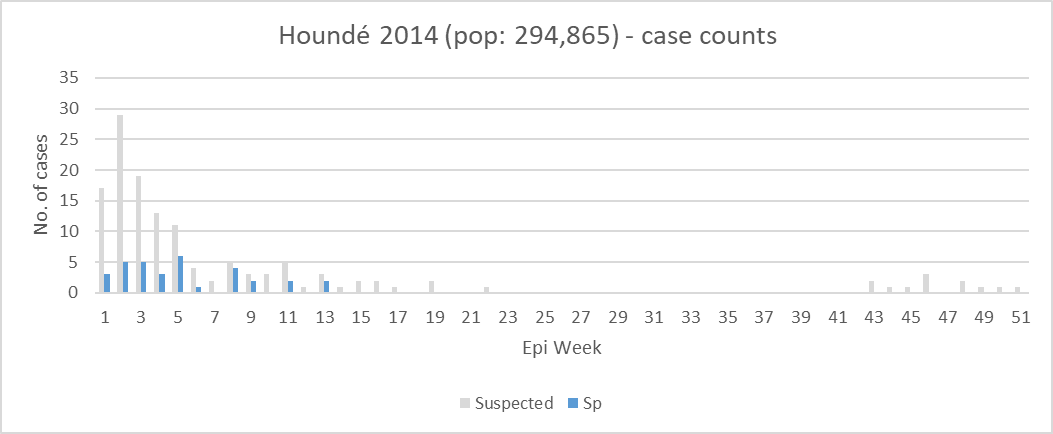

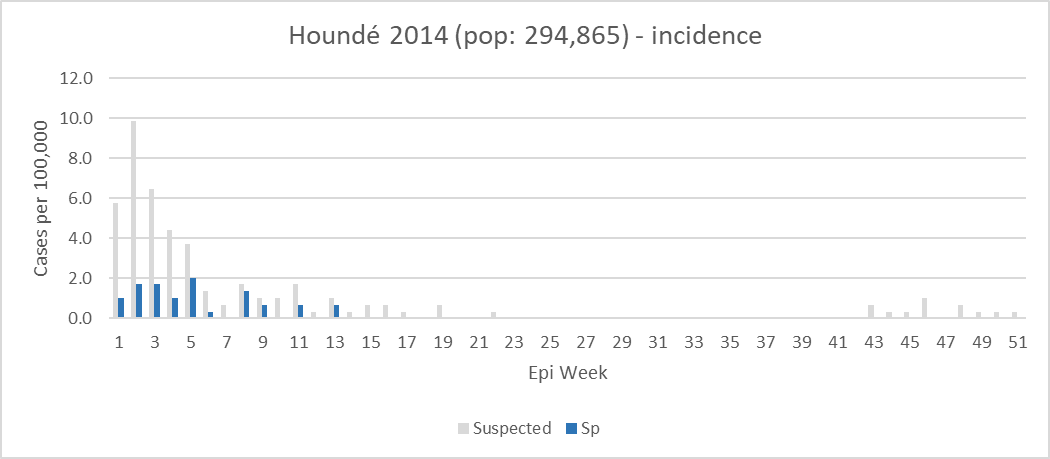

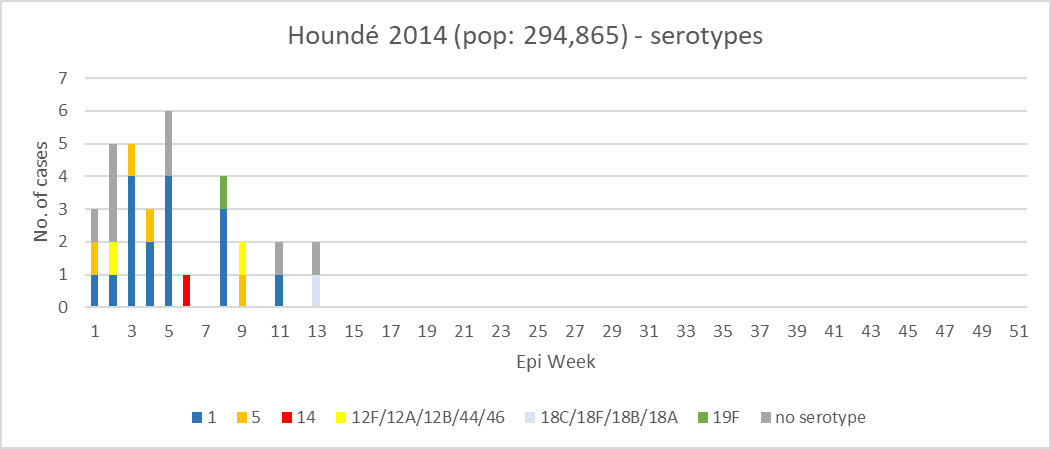

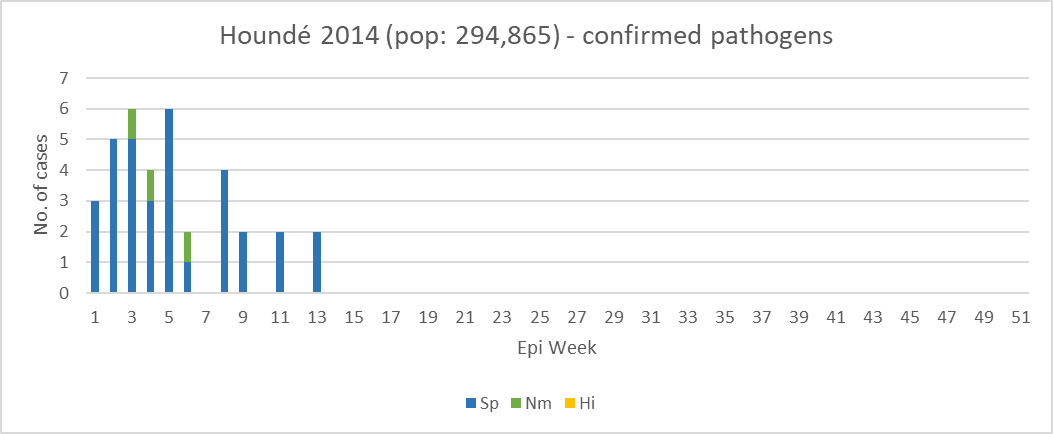


# Kaya 2017, Epidemiologic week 7


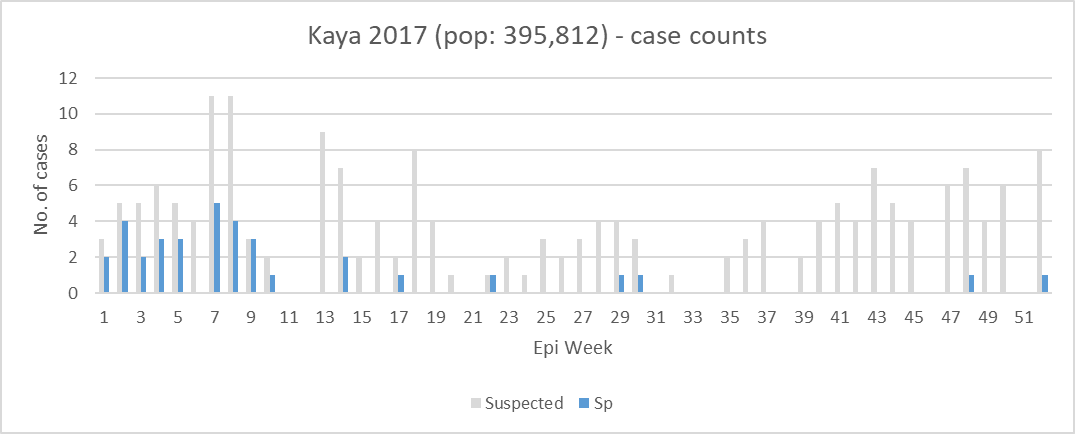

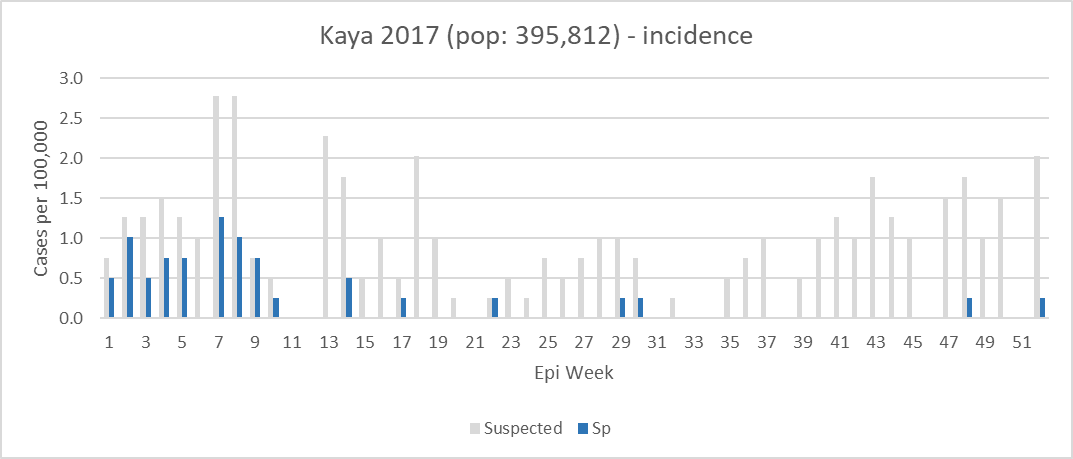

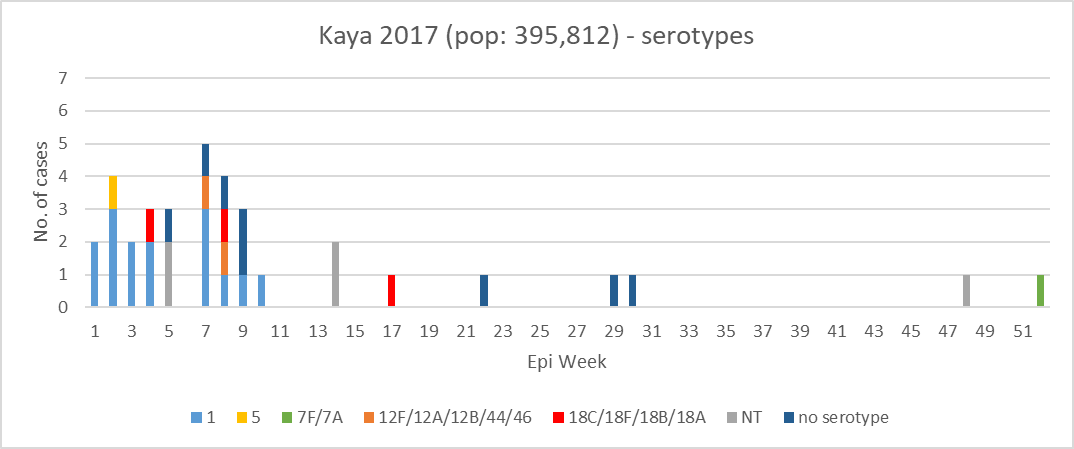

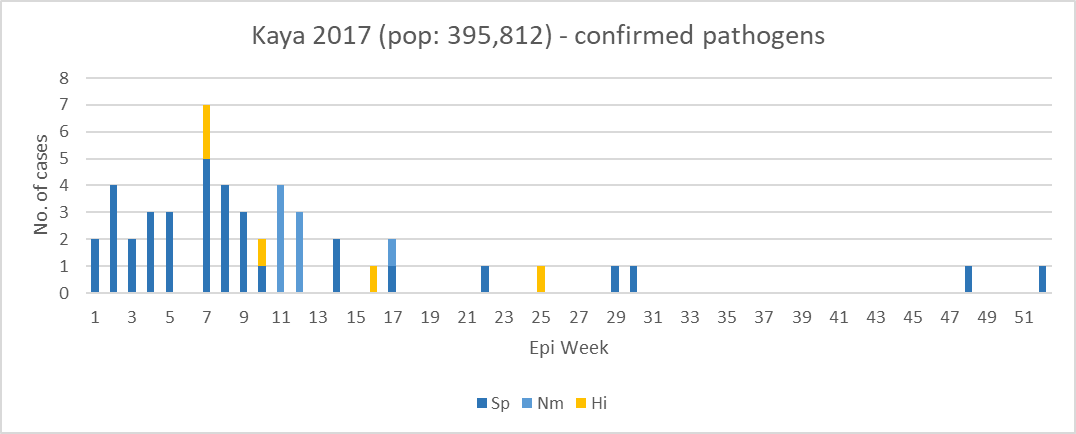


# Titao 2017, Epidemiologic week 4


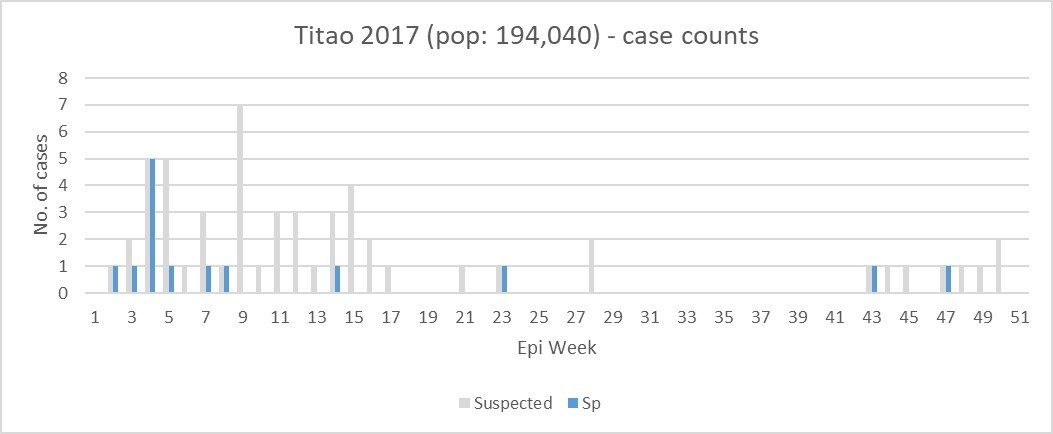

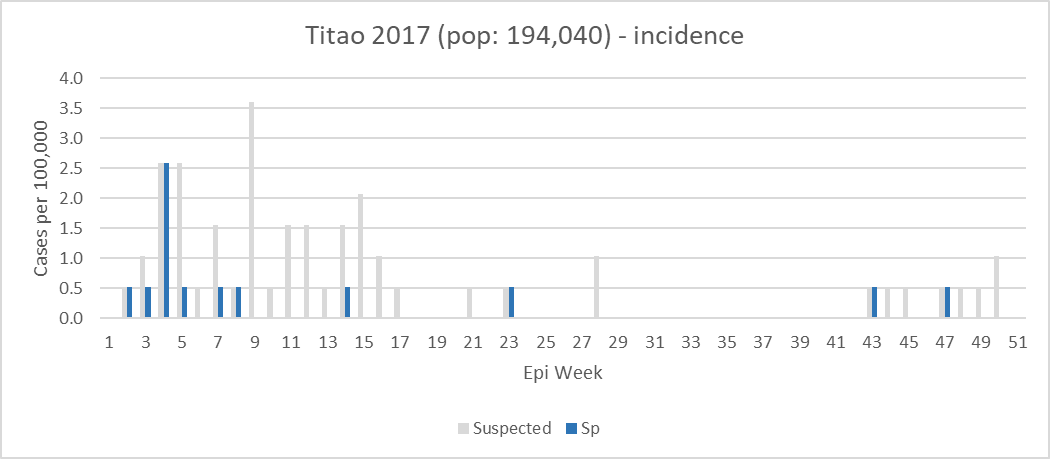

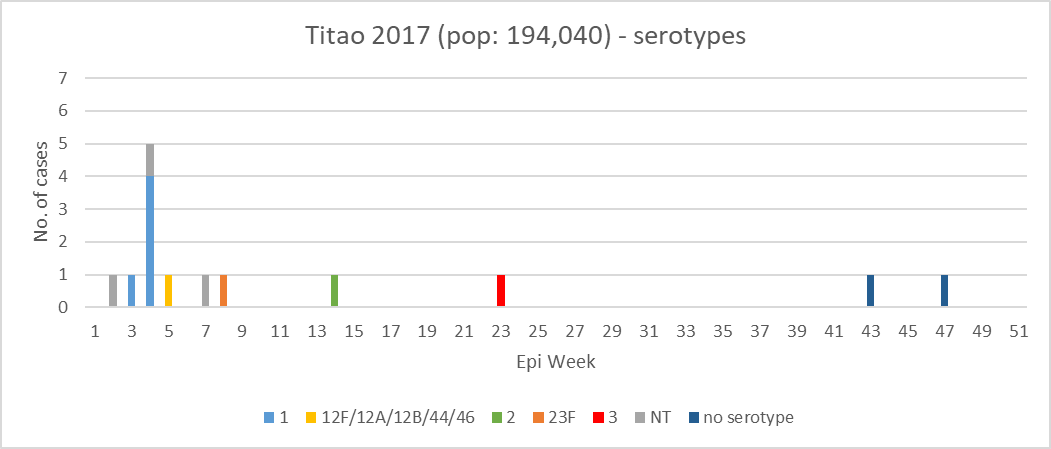

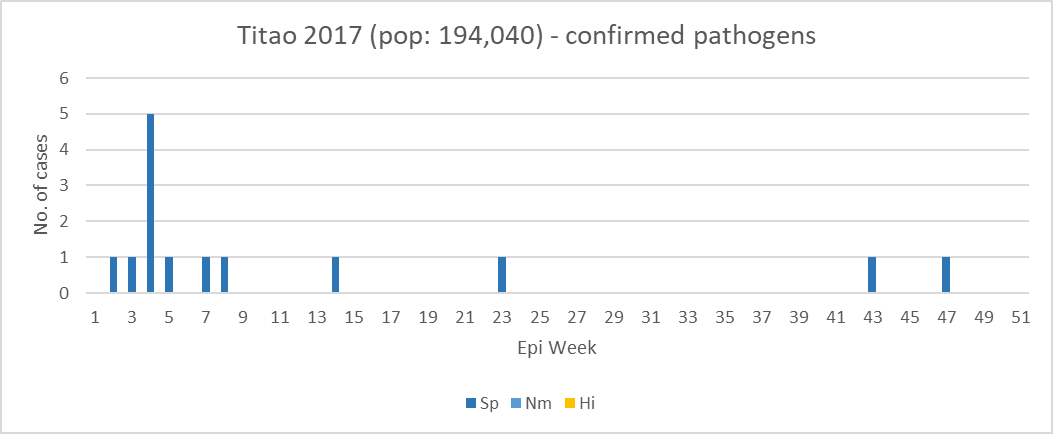

Supplement: Supplementary data 1 [file mmc1.docx]
